# Synthesis, characterization and evaluation of 1,3,5-triazine aminobenzoic acid derivatives for their antimicrobial activity

**DOI:** 10.1186/s13065-017-0267-3

**Published:** 2017-05-10

**Authors:** Khadijah M. Al-Zaydi, Hosam H. Khalil, Ayman El-Faham, Sherine N. Khattab

**Affiliations:** 1 Department of Chemistry, Faculty of Sciences-AL Faisaliah, King Abdulaziz University, Jeddah, P.O. Box 50918, Jeddah, 21533 Kingdom of Saudi Arabia; 2 Department of Chemistry, Faculty of Science, Alexandria University, P.O. Box 426, Ibrahimia, Alexandria, 21321 Egypt

**Keywords:** 1,3,5-Triazine derivatives, 4-Aminobenzoic acid, Morpholine, Piperidine, Aniline, Benzylamine, Diethylamine, Microwave irradiation, Antimicrobial activity

## Abstract

**Background:**

Replacement of chloride ions in cyanuric chloride give several variants of 1,3,5-triazine derivatives which were investigated as biologically active small molecules. These compounds exhibit antimalarial, antimicrobial, anti-cancer and anti-viral activities, among other beneficial properties. On the other hand, treatment of bacterial infections remains a challenging therapeutic problem because of the emerging infectious diseases and the increasing number of multidrug-resistant microbial pathogens. As multidrug-resistant bacterial strains proliferate, the necessity for effective therapy has stimulated research into the design and synthesis of novel antimicrobial molecules.

**Results:**

1,3,5-Triazine 4-aminobenzoic acid derivatives were prepared by conventional method or by using microwave irradiation. Using microwave irradiation gave the desired products in less time, good yield and higher purity. Esterification of the 4-aminobenzoic acid moiety afforded methyl ester analogues. The *s*-triazine derivatives and their methyl ester analogues were fully characterized by FT-IR, NMR (^1^H-NMR and ^13^C-NMR), mass spectra and elemental analysis. All the synthesized compounds were evaluated for their antimicrobial activity. Some tested compounds showed promising activity against *Staphylococcus aureus* and *Escherichia coli.*

**Conclusions:**

Three series of mono-, di- and trisubstituted s-triazine derivatives and their methyl ester analogues were synthesized and fully characterized. All the synthesized compounds were evaluated for their antimicrobial activity. Compounds (**10**), (**16**), (**25**) and (**30**) have antimicrobial activity against *S. aureus* comparable to that of ampicillin, while the activity of compound (**13**) is about 50% of that of ampicillin. Compounds (**13**) and (**14**) have antimicrobial activity against *E. coli* comparable to that of ampicillin, while the activity of compounds (**9**–**12**) and (**15**) is about 50% of that of ampicillin. Furthermore, minimum inhibitory concentrations values for clinical isolates of compounds (**10**), (**13**), (**14**), (**16**), (**25**) and (**30**) were measured. Compounds (**10**) and (**13**) were more active against *MRSA* and *E. coli* than ampicillin. Invitro cytotoxicity results revealed that compounds (**10**) and (**13**) were nontoxic up to 250 µg/mL (with SI = 10) and 125 µg/mL (with SI = 5), respectively.

**Graphical abstract:**
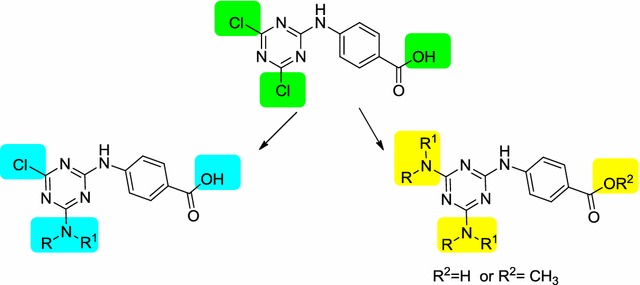
Three series of mono-, di- and trisubstituted s-triazine derivatives and their methyl ester analogues were synthesized and evaluated for their antimicrobial activity. Several compounds have antimicrobial activity against *S. aureus* and *E. coli* comparable to that of ampicillin.

**Electronic supplementary material:**

The online version of this article (doi:10.1186/s13065-017-0267-3) contains supplementary material, which is available to authorized users.

## Background

Sophisticated s-triazine derivatives can be easily prepared from the cheap and readily available 2,4,6-trichloro-1,3,5-triazine (cyanuric chloride) **1** [1–3]. Replacement of chloride ions in cyanuric chloride give several variants of 1,3,5-triazine derivatives, which were investigated as biologically active small molecules [4–8]. These compounds exhibit antimalarial [9–16], antimicrobial [17–25], anti-cancer [26–31] and anti-viral activities [32], among other beneficial properties. On the other hand, treatment of bacterial infections remains a challenging therapeutic problem because of emerging infectious diseases and the increasing number of multidrug-resistant microbial pathogens [33]. As multidrug-resistant bacterial strains proliferate, the necessity for effective therapy has stimulated research into the design and synthesis of novel antimicrobial molecules [34–42].

In this study, we prepared several 1,3,5-triazine derivatives by replacing one, two, or three chloride ions of cyanuric chloride with different *N*-nucleophiles (including 4-aminobenzoic acid, methyl p-aminobenzoate, aniline, benzylamine, diethylamine, morpholine and piperidine) and evaluated their antimicrobial activity.

## Results and discussion

### Chemistry

Cyanuric chloride (**1**) is definitely an excellent starting compound for the straight forward preparation of highly structured multitopic molecules [1]. The first substitution is exothermic; therefore, the temperature of the reaction mixture has to be maintained at 0 °C. The substitution of the second chloride can be performed at room temperature. Finally, the third position is functionalized under reflux of the solvent. As a result, a careful control of the reaction temperature during the substitution reactions will allow the synthesis of 2,4,6-trisubstituted-1,3,5-triazines by the sequential and very selective addition of amine nucleophiles [1, 43].

**Scheme 1:**
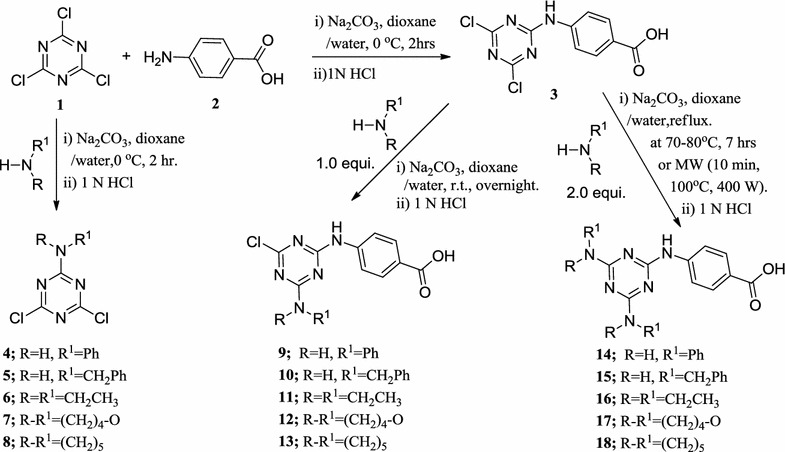
Synthesis of mono-, di- and trisubstituted*s*-triazine derivatives **3** *–* **18**

A monosubstituted 1,3,5-triazine series was prepared selectively by substituting one chloride ion of cyanuric chloride (1) with 4-aminobenzoic acid (**2**), aniline, benzylamine, diethylamine, morpholine or piperidine in the presence of sodium carbonate as an acid scavenger of the liberated hydrochloric acid, in an ice-bath, to afford products 4-((4,6-dichloro-1,3,5-triazin-2yl)amino)benzoic acid (**3**), 4,6-dichloro-*N*-phenyl-1,3,5-triazin-2-amine (**4**), *N*-benzyl-4,6-dichloro-1,3,5-triazin-2-amine (**5**), 4,6-dichloro-*N,N*-diethyl-1,3,5-triazin-2-amine (**6**), 4-(4,6-dichloro-1,3,5-triazin-2-yl)morpholine (**7**), and 2,4-dichloro-6-(piperidin-1-yl)-1,3,5-triazine (**8**), respectively (Scheme 1). The structure of the products was confirmed using NMR (^1^H and ^13^C), IR and elemental analysis.

In addition, a series of disubstituted 1,3,5-triazine was prepared by replacement of the second chloride ion of 4-((4,6-dichloro-1,3,5-triazin-2-yl)amino)benzoic acid (**3**) with aniline, benzylamine, diethylamine, morpholine, or piperidine, respectively. The reaction mixture was stirred overnight at room temperature in presence of sodium carbonate to give 4-((4-chloro-6-(phenylamino)-1,3,5-triazin-2-yl)amino)benzoic acid (**9**), 4-((4-(benzylamino)-6-chloro-1,3,5-triazin-2-yl)amino)benzoic acid **(10**), 4-((4-chloro-6-(diethylamino)-1,3,5-triazin-2-yl)amino)benzoic acid (**11)**, 4-((4-chloro-6-morpholino-1,3,5-triazin-2-yl)amino)benzoic acid (**12**), and 4-((4-chloro-6-(piperidin-1-yl)-1,3,5-triazin-2-yl)amino)benzoic acid (**13**), respectively (Scheme 1). The structure of the products was confirmed using NMR (^1^H and ^13^C), IR, mass spectra and elemental analysis.

**Fig. 1:**
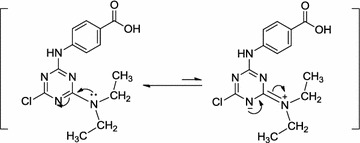
Restricted rotation around C_6_–N bond due to resonance

The ^1^H-NMR of compound (**11**) in DMSO-*d*_*6*_ showed that the two ethyl groups of the diethylamino moiety are not equivalent, as indicated from their chemical shift values, where the two methyl groups are observed at 1.09 and 1.15 ppm as two triplets and the methylene groups are observed at the range 3.50–3.53 ppm as a multiplet. These observations indicate that the two ethyl groups are found in different electronic environments. This fact can be attributed to restricted rotation around the C_6_–N bond due to resonance that gives this bond some double bond character [44]. The difference in the chemical shifts of ethyl groups are probably due to differences in the field effects (anisotropic effects) of the benzoyl group of 4-aminobenzoic acid substituents on C_2_ on either ethyl groups (Fig. 1). The aromatic protons appear as two doublets at chemical shifts 7.79 and 7.86 ppm. The two D_2_O exchangeable protons (NH and OH) are observed at 10.34 and 12.69 ppm, respectively. The previous prediction was confirmed by the ^13^C-NMR spectrum of compound (**11**), where the two methyl carbons appear as two peaks at 13.01 and 13.47 ppm and the two methylene carbons are observed at 42.01 and 42.47 ppm.

Furthermore, C_2_-symmetrical 1,3,5-triazine tripod series were prepared by replacing the two chloride ions of 4-((4,6-dichloro-1,3,5-triazin-2-yl)amino)benzoic acid (**3)** with two equivalents of aniline, benzylamine, diethylamine, morpholine, or piperidine, respectively. The reaction proceeded at 70–80 °C in dioxane/water solvent mixture using sodium carbonate as a base by the conventional method; or by using microwave irradiation and employing a multimode reactor (Synthos 3000, Aton Paar GmbH, 1400 W maximum magnetron) to produce the corresponding products: 4-((4,6-bis(phenylamino)-1,3,5-triazin-2-yl)amino)benzoic acid (**14**), 4-((4,6-bis(benzylamino)-1,3,5-triazin-2-yl)amino)benzoic acid (**15**), 4-((4,6-bis(diethylamino)-1,3,5-triazin-2-yl)amino)benzoic acid (**16**), 4-((4,6-dimorpholino-1,3,5-triazin-2-yl)amino)benzoic acid (**17**), 4-((4,6-di(piperidin-1-yl)-1,3,5-triazin-2-yl)amino)benzoic acid (**18**), respectively (Scheme 1). Using microwave irradiation produced the desired products in less time, good yield and higher purity. The structures of the products were confirmed using NMR (^1^H and ^13^C), IR, mass spectra and elemental analysis. As a prototype of this series, in the ^1^H-NMR of compound **17,** the methylene group protons of morpholine moiety appear as multiplet at the chemical shift range of 3.60–3.69 ppm, and the *p*-disubstituted benzene ring protons appear as two doublets at chemical shifts 7.73 and 7.83 ppm. The N–H proton appears at the chemical shift range of 9.74–9.87 ppm as a broad D_2_O exchangeable peak.

**Scheme 2:**
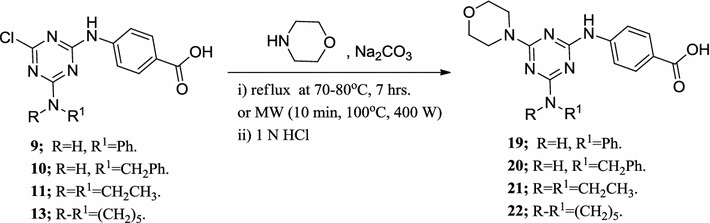
Synthesis of tripod morpholino-*s*-triazine derivatives **19**–**22**

**Fig. 2:**
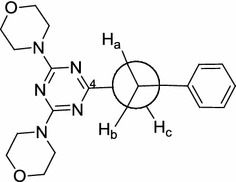
Newman projection formula of *N*-(4-benzylamino-6-morpholino-1,3,5-triazin-2-yl) aminobenzoic acid **20**

Similarly, another tripod series of 1,3,5-triazine derivatives (**19**–**22**) was synthesized, which differed only in one position of the triazine nucleus, but contained 4-aminobenzoic acid and morpholino moieties in all members of this series. Chloride ion of compounds (**9**–**11**) and (**13**) was replaced with one equivalent of morpholine at 70–80 °C in a dioxane/water solvent mixture using sodium carbonate as a base by the conventional method, or by using microwave irradiation, employing a multimode reactor (Synthos 3000, Aton Paar GmbH, 1400 W maximum magnetron) to give the corresponding products (Scheme 2). Using microwave irradiation produced the desired products in less time, good yield and higher purity. The structures of the products were confirmed using NMR (^1^H and ^13^C), IR and elemental analyses. As a prototype of this series, in the ^1^H-NMR of compound (**20**), the eight methylene protons of morpholine appear as multiplet at a chemical shift range of 3.57–3.74 ppm, while the benzyl methylene group protons appear as multiplet at 4.45–4.50 ppm. The nine aromatic protons appear as multiplet at chemical shift ranges of 7.18–7.71 and 7.69–7.85 ppm. The N–H proton (D_2_O exchangeable) appears as multiplet at 9.49–9.73 ppm due to its coupling with the adjacent methylene protons. The O–H proton (D_2_O exchangeable) is observed at 12.40 ppm as a singlet peak. The multiplet appearance of benzyl methylene protons indicates that the two protons are not equivalent (enantiotopic protons). From the conformational point of view, using the Newman projection formula (Fig. 2), H_a_ and H_b_ are not equivalent due to restricted rotation around the C_4_–N bond (Fig. 1); as a result, they can couple via germinal coupling depending on the H_a_CH_b_ angle in addition to their vicinal coupling of the NH_c_ proton, as shown by the staggered conformation of (**20**) (Fig. 2). This behavior also explains the multiplet appearance of the N–H_c_ proton.

**Scheme 3:**
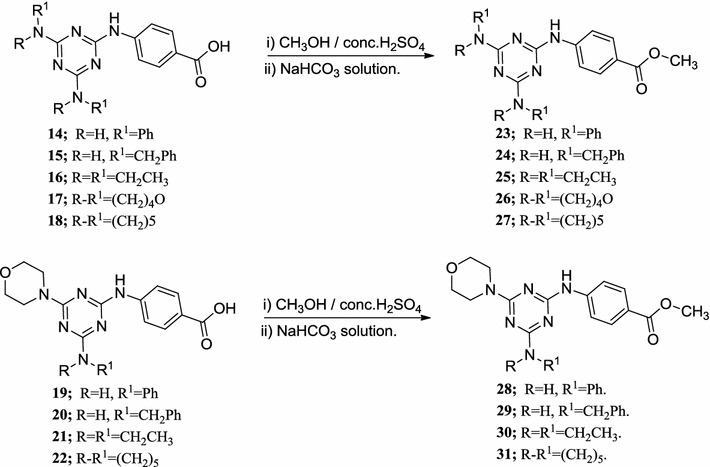
Synthesis of methyl ester of C_2_—symmetrical and morpholino 1,3,5-triazine tripod derivatives **23**–**31**

Furthermore, esterification of the previously prepared compounds (**14**–**22**) afforded compounds (**23**–**31**) (Scheme 3). The structure of the products was confirmed using NMR (^1^H and ^13^C), IR, mass spectra and elemental analysis.

### Antimicrobial activity

The synthesized compounds (**9**–**31**) have been evaluated for their antimicrobial activity against *E. coli* representing Gram-negative bacteria, *S. aureus* representing Gram-positive bacteria and *C. albicans* representing fungi. Microdilution susceptibility test in Müller–Hinton Broth (Oxoid) and Sabouraud Liquid Medium (Oxoid) were used for the determination of antibacterial and antifungal activity [45]. The minimal inhibitory concentration (MIC) values listed in Table 1 showed that all tested compounds have lower antifungal activity than clotrimazole (Canesten^®^, Bayer).

**Table 1:** Minimal inhibitory concentration (MIC) of test compounds in μg/mL

Test compound	*E. coli*	*S. aureus*	*C. albicans*	Test compound	*E. coli*	*S. aureus*	*C. albicans*
**Ampicillin**	25	12.5	–	**20**	100	>200	>200
**Clotrimazole**	–	–	12.5	**21**	100	50	>200
**9**	50	100	>200	**22**	100	100	>200
**10**	50	**12.5**	>200	**23**	100	100	>200
**11**	50	100	>200	**24**	100	100	>200
**12**	50	50	>200	**25**	100	**12.5**	>200
**13**	**25**	**25**	>200	**26**	100	100	>200
**14**	**25**	100	>200	**27**	100	100	>200
**15**	50	>200	>200	**28**	100	>200	>200
**16**	100	**12.5**	>200	**29**	100	>200	>200
**17**	100	100	>200	**30**	100	**12.5**	>200
**18**	100	>200	>200	**31**	100	100	>200
**19**	>200	100	>200				

The synthesized compounds are more active against *S. aureus* and *E. coli*. Compounds (**10**), (**16**)**, (25**) and (**30**) have antimicrobial activity against *S. aureus* comparable to that of ampicillin, while the activity of compound (**13**) is about 50% of that of ampicillin. Compounds (**13**) and (**14**) have antimicrobial activity against *E. coli* comparable to that of ampicillin, while the activity of compounds (**9**–**12**) and (**15**) is about 50% of that of ampicillin.

Furthermore, minimum inhibitory concentrations (MIC µg/mL) values for clinical isolates of compounds (**10**), (**13**), (**14**), (**16**)**, (25**) and (**30**) were also tested and listed in Table 2. Compounds (**10**) and (**13**) were more active against *MRSA* and *E. coli* than ampicillin.

**Table 2:** Minimum inhibitory concentrations (MIC µg/mL) for clinical isolates of compounds (**10**), (**13**), (**14**), (**16**)**, (25**) and (**30**)

Test compound	*MRSA*	*E. coli*
**Ampicillin**	>200	>200
**10**	**25**	100
**13**	100	**25**
**14**	>200	100
**16**	100	>200
**25**	100	100
**30**	>200	>200

*MRSA* methicillin-resistant *Staphylococcus aureus*

Invitro cytotoxicity of the most active compounds (**10**) and (**13**) were carried out with 5ero cell line using Mosmann method with certain modifications as described in the literature [46]. 50% cytotoxic concentration (CC_50_) expressed in µg/mL and selectivity index (SI) values were listed in Table 3. The results revealed that the test compounds (**10**) and (**13**) were nontoxic up to 250 µg/mL (with SI = 10) and 125 µg/mL (with SI = 5), respectively.

**Table 3:** CC_50_ values and selectivity index (SI) of the most active compounds on normal VERO

Test compound	(CC_50_)^a^ (µg/mL)	Selectivity index (SI)^b^
**10**	250	10
**13**	125	5

^a^CC_50_ is the concentration of compound required to kill 50% of the fibroblast cells

^b^The selectivity index (SI) was calculated using the formula, SI = CC_50_/MIC

## Experimental section

### Chemistry

Solvents and reagents were purchased from Sigma-Aldrich. Unless otherwise stated, the normal workup from organic solvent involved drying over Na_2_SO_4_ and rotary evaporation. TLC was performed using aluminum-backed Merck Silica Gel 60 F-254 plates using suitable solvent systems with spots being visualized by a Spectroline UV Lamp (254 or 365 nm) or I_2_ vapor. Melting points were obtained in open capillary tubes using a MEL-Temp II melting point apparatus and are uncorrected. Microwave experiments were performed using a multimode reactor (Synthos 3000, Aton Paar GmbH, 1400 W maximum magnetron). Infrared spectra (IR) were recorded on a Perkin-Elmer 1600 series Fourier transform instrument as KBr pellets. The absorption bands (max) are given in wave numbers (cm^−1^). Nuclear magnetic resonance (NMR) spectra (^1^H-NMR and ^13^C-NMR) were recorded on a JEOL 500 MHz spectrometer at ambient temperature. Chemical shifts are reported in parts per million (ppm) and are referenced relative to residual solvent (e.g. CHCl_3_ at δH 7.26 ppm for CDCl_3_, DMSO at δH 2.50 ppm for DMSO-d_6_). Spin multiplicities are represented by the following signals: singlet (s), broad singlet (br s), doublet (d), doublet of doublets (dd), triplet (t), doublet of triplets (dt), quartet (q), sextet (sex) and multiplet (m). Elemental analyses were performed on a Perkin-Elmer 2400 elemental analyzer, and the values found were within ±0.3% of the theoretical values. Mass spectra (MS) were recorded on a QP 1000EX/MS Shimatzu Corp by using electron impact (EI) at 70 eV. The antimicrobial activity and invitro cytotoxicity were carried at the lab of Prof. Adnan Bekhit, Faculty of Pharmacy, Alexandria University.

#### General procedure for the synthesis of 2-substituted-4,6-dichloro-1,3,5-triazine derivatives (3–8)

To a solution of cyanuric chloride (1.01 g, 5.5 mmol) in methylene chloride (10 mL), amine (5 mmol) and sodium carbonate (1.06 g, 10 mmol) were added. The mixture was vigorously stirred at 0–5 °C for 3 h. The precipitate was filtered and washed with methylene chloride. The precipitate was dissolved in small amount of water. The solution was neutralized with 1 N HCl, and the formed precipitate was filtered (Additional file 1).

##### *N*-(4,6-dichloro-1,3,5-triazin-2-yl)aminobenzoic acid (3)

The product was obtained as white solid, 1.36 g (95.4%) yield; mp > 360 °C [Lit mp > 350 °C] [47]. ^1^H-NMR (500 MHz, DMSO-d_6_): δ 7.70 (d, 2H, *J* = 8.4 Hz, Ar–H), 7.88 (d, 2H, *J* = 8.4 Hz, Ar–H), 10.88 (s, 1H, NH, D_2_O exchangeable).

##### 4,6-dichloro-*N*-phenyl-1,3,5-triazin-2-amine (4) [48]

The product was obtained as white solid, 1.11 g (92.1%) yield; mp: 235–238 °C.

##### *N*-benzyl-4,6-dichloro-1,3,5-triazin-2-amine (5) [49]

The product was obtained as white solid, 1.16 g (90.8%) yield; mp: 232–234 (dec.) °C; IR (KBr): 3650–2700 (br, OH, acid), 3268 (NH, amine), 1686 (CO, acid) cm^−1^; ^1^H-NMR (500 MHz, DMSO-d_6_): δ 4.49–4.53 (m, 2H, CH_2_), 7.28–7.31 (m, 5H, Ar–H), 9.60 (t, 1H, *J* = 6.1 Hz, NH, D_2_O exchangeable), 11.15 (s, 1H, OH, D_2_O exchangeable).

##### 4,6-dichlro-*N,N*-diethyl-1,3,5-triazin-2-amine (6) [50]

The product was obtained as white solid, 1.05 g (94.6%) yield; mp: 241–244 °C; ^1^H-NMR (500 MHz, DMSO-d_6_): δ 1.09–1.11 (m, 6H, 2CH_3_), 3.51–3.55 (m, 4H, 2CH_2_), 11.15 (s, 1H, OH, D_2_O exchangeable).

##### 4-(4,6-dichloro-1,3,5-triazin-2-yl)morpholine (7)

The product was obtained as white solid, 1.03 g (87.3%) yield; mp: 157–158 °C [Lit mp 154–156 °C] [16];.^1^H-NMR (500 MHz, CDCl_3_): δ 3.69 (t, 4H, *J* = 5.4 Hz, 2× CH_2_N), δ 3.73 (t, 4H, *J* = 5.4 Hz, 2× CH_2_O).

##### 2,4-dichloro-6-(piperidin-1-yl)-1,3,5-triazine (**8**)

The product was obtained as white solid, 1.04 g (89.2%) yield; mp: 143–145 °C [Lit mp 176–178 °C] [16]; ^1^H-NMR (500 MHz, CDCl_3_): δ 1.60–1.65 (m, 4H, 2CH_2_), 1.69–1.72 (m, 2H, CH_2_), 3.80 (t, 4H, *J* = 6.1 Hz, 2CH_2_–N).

#### General procedure for the synthesis of 4-((4-chloro-6-substituted-1,3,5-triazin-2-yl)amino)benzoic acid derivatives (9–13)

To a solution of *N*-(4,6-dichloro-1,3,5-triazin-2-yl)aminobenzoic acid **3** (2.0 g, 7.0 mmol) and sodium carbonate (1.78 g, 16.8 mmol) in distilled water (20 mL), a solution of amine (8.4 mmol) in dioxane (5 mL) was added while stirring. The reaction mixture was stirred overnight at room temperature. The reaction mixture was neutralized with 1 N HCl. The formed precipitate was filtered and washed with water.

##### 4-((4-chloro-6-(phenylamino)-1,3,5-triazin-2-yl)amino)benzoic acid (9)

The product was obtained as white solid, 2.38 g (99.5%) yield; mp: 292-295 (dec.) ^o^C; IR (KBr): 3700–2500 (br, OH, acid), 3278 (NH, amine), 1691 (CO, acid) cm^−1^; ^1^H-NMR (500 MHz, DMSO-d_6_): δ 6.98–7.35 (m, 4H, Ar–H), 7.62–7.97 (m, 5H, Ar–H), 9.38–9.73 (m, 1H, NH, D_2_O exchangeable), 10.25–10.56 (m, 1H, NH, D_2_O exchangeable), 12.72 (s, 1H, OH, D_2_O exchangeable); ^13^C-NMR (125 MHz, DMSO-d_6_): 120.32, 121.14, 128.94, 129.15, 129.36, 130.60, 140.23, 144.96, 164.42, 164.52, 167.47, 167.68. Elemental analysis calcd. for C_16_H_12_ClN_5_O_2_: C, 56.23; H, 3.54; Cl, 10.37; N, 20.49. Found: C, 56.20; H, 3.59; Cl, 10.33; N, 20.52.

##### 4-((4-(benzylamino)-6-chloro-1,3,5-triazin-2-yl)amino)benzoic acid (10)

The product was obtained as white solid, 2.48 g (99.6%) yield; mp: 272–275 (dec.) ^o^C; IR (KBr): cm^−1^; ^1^H-NMR (500 MHz, DMSO-d_6_): δ 4.48–4.52 (m, 2H, CH_2_), 7.20–7.31 (m, 4H, Ar–H), 7.76–7.84 (m, 5H, Ar–H), 8.70–8.83 (m, 1H, NH, D_2_O exchangeable), 10.31–10.35 (m, 1H, NH, D_2_O exchangeable), 12.61 (s, 1H, OH, D_2_O exchangeable); ^13^C-NMR (125 MHz, DMSO-d_6_): 44.2, 119.69, 125.01, 127.31, 127.87, 128.94, 130.62, 166.00, 166.18, 167.51, 168.60. Elemental analysis calcd. for C_17_H_14_ClN_5_O_2_:C, 57.39; H, 3.97; Cl, 9.96; N, 19.68. Found: C, 57.29; H, 4.03; Cl, 9.98; N, 19.64.

##### 4-((4-chloro-6-(diethylamino)-1,3,5-triazin-2-yl)amino)benzoic acid (11)

The product was obtained as white solid, 2.04 g (90.6%) yield; mp: 220–222 (dec.) ^o^C; IR (KBr): 3700–2500 (br, OH, acid), 3285 (NH, amine), 1689 (CO, acid) cm^−1^; ^1^H-NMR (500 MHz, DMSO-d_6_): δ 1.09 (t, 3H, *J* = 6.9 Hz, CH_3_), 1.15 (t, 3H, *J* = 6.9 Hz, CH_3_), 3.50–3.53 (m, 4H, 2CH_2_), 7.79 (d, 2H, *J* = 8.4 Hz, Ar–H), 7.86 (d, 2H, *J* = 8.4 Hz, Ar–H), 10.34 (s, 1H, NH, D_2_O exchangeable), 12.69 (s, 1H, OH, D_2_O exchangeable); ^13^C-NMR (125 MHz, DMSO-d_6_): 13.01, 13.47, 42.01, 42.47, 119.50, 125.03, 130.73, 143.78, 163.87, 164.12, 167.51, 168.83. EIMS (m/z): 321.139 (M^+^); elemental analysis calcd. for C_14_H_16_ClN_5_O_2_: C, 52.26; H, 5.01; Cl, 11.02; N, 21.77. Found: C, 52.30; H, 5.05; Cl, 10.98; N, 21.80.

##### 4-((4-chloro-6-morpholino-1,3,5-triazin-2-yl)amino)benzoic acid (12)

The product was obtained as white solid, 2.14 g (91.1%) yield; mp: 308–311 (dec.)  °C; IR (KBr): 3700–2500 (br, OH, acid), 3413(NH, amine), 1684 (CO, acid) cm^−1^; ^1^H-NMR (500 MHz, DMSO-d_6_): δ 3.58–3.62 (m, 4H, 2CH_2_), 3.65–3.69 (m, 4H, 2CH_2_), 7.77–7.86 (m, 4H, Ar–H), 10.41 (s, 1H, NH, D_2_O exchangeable), 12.61 (s, 1H, OH, D_2_O exchangeable); ^13^C-NMR (125 MHz, DMSO-d_6_): 43.91, 66.54, 119.02, 125.26, 130.81, 143.46, 164.50, 165.17, 167.49, 167.66. Elemental analysis calcd. for C_14_H_14_ClN_5_O_3_: C, 50.08; H, 4.20; Cl, 10.56; N, 20.86. Found: C, 50.17; H, 4.15; Cl, 10.51; N, 20.98.

##### 4-((4-chloro-6-(piperidin-1-yl)-1,3,5-triazin-2-yl)amino)benzoic acid (13)

The product was obtained as white solid, 2.11 g (90.3%) yield; mp: 284–287 (dec.) ^o^C; IR (KBr): 3700–2600 (br, OH, acid), 3269 (NH, amine), 1689 (CO, acid) cm^−1^; ^1^H-NMR (500 MHz, DMSO-d_6_): δ 0.85–0.88 (m, 3H, pip), 1.51–1.54 (m, 2H, CH_2_-pip), 3.18–3.24 (m, 3H, pip), 7.80–7.84 (m, 4H, Ar–H), 10.26–10.37 (m, 1H, NH, D_2_O exchangeable), 12.72 (s, 1H, OH, D_2_O exchangeable); ^13^C-NMR (125 MHz, DMSO-d_6_): 11.92, 22.36, 42.96, 119.63, 125.05, 130.68, 143.80, 164.27, 165.91, 167.51, 168.43. Elemental analysis calcd. for C_15_H_16_ClN_5_O_2_:C, 53.98; H, 4.83; Cl, 10.62; N, 20.98. Found: C, 53.91; H, 4.89; Cl, 10.56; N, 21.03.

#### General procedure for the synthesis of 4-((4,6-disubstituted-1,3,5-triazin-2-yl)amino)benzoic acid derivatives (14-18)

##### Method A

Conventional procedure: To a solution of *N*-(4,6-dichloro-1,3,5-triazin-2-yl)aminobenzoic acid **3** (0.71 g, 2.5 mmol) and sodium carbonate (0.95 g, 9.0 mmol) in distilled water (20 mL), a solution of amine (6.25 mmol) in dioxane (5 mL) was added while stirring. The reaction mixture was stirred at room temperature for 2 h then refluxed at 70–80 °C for 8–10 h. The reaction mixture was neutralized with 1 N HCl after cooling. The corresponding crude products were filtered, dried, and recrystallized from ethanol.

##### Method B

Microwave-irradiation: Employing a multimode reactor (Synthos 3000, Aton Paar GmbH, 1400 W maximum magnetron), the initial step was conducted with 4-Teflon vessels rotor (MF 100) that allow processing four reactions under the same conditions. Each vessel has *N*-(4,6-dichloro-1,3,5-triazin-2-yl)aminobenzoic acid **3** (0.71 g, 2.5 mmol) and sodium carbonate (0.95 g, 9.0 mmol) mixed with the appropriate amine (6.25 mmol) in 3 mL dioxane/water (1:1). The individual vessels were purged with nitrogen gas for 5 min and then were placed in the corresponding rotor, fixed by screwing down the upper rotor place, and finally the rotor was closed with a protective hood. The vessels were heated for 5 min at 100 °C and held at the same temperature for a further 5 min (~2 bar pressure, 400 W). Cooling was accomplished by a fan (5 min). The reaction mixture was neutralized with 1 N HCl after cooling. The corresponding crude products were filtered, dried, and recrystallized from ethanol (Additional file 1).

##### 4-((4,6-bis(phenylamino)-1,3,5-triazin-2-yl)amino)benzoic acid (14)

The product was obtained as white solid, *Method A* 0.75 g (75.3%) yield; *Method B* 0.91 g (91%) yield; mp: 318–320 °C; IR (KBr): 3600–2700 (br, OH, acid), 3413 (NH, amine), 1686 (CO, acid) cm^−1^; ^1^H-NMR (500 MHz, DMSO-d_6_): δ 7.27–7.83 (m, 14H, Ar–H), 9.34–9.89 (m, 3H, NH, D_2_O exchangeable), 12.53 (s, 1H, OH, D_2_O exchangeable); ^13^C-NMR (125 MHz, DMSO-d_6_): 119.48, 121.14, 122.83, 124.07, 128.94, 130.54, 140.27, 144.96, 164.52, 164.63, 167.76. Elemental analysis calcd. for C_22_H_18_N_6_O_2_: C, 66.32; H, 4.55; N, 21.09. Found: C, 66.12; H, 4.65; N, 21.01.

##### 4-((4,6-bis(benzylamino)-1,3,5-triazin-2-yl)amino)benzoic acid (15)

The product was obtained as white solid, *Method A* 0.85 g (79.2%) yield; *Method B* 0.95 g (88.5%) yield; mp: 308–310 °C; IR (KBr): 3600–2800 (br, OH, acid), 3441 (NH, amine), 1677 (CO, acid) cm^−1^; ^1^H-NMR (500 MHz, DMSO-d_6_): δ 4.42–4.49 (m, 4H, 2CH_2_), 7.18–7.77 (m, 16H, 14 Ar–H, 2NH), 9.25–9.31 (m, 1H, NH, D_2_O exchangeable), 11.97 (s, 1H, OH, D_2_O exchangeable); ^13^C-NMR (125 MHz, DMSO-d_6_): 43.93, 118.77, 127.03, 127.41, 127.62, 127.95, 128.71, 130.43. 141.07, 164.61, 166.18, 166.27, 167.95. EIMS (m/z): 426.055 (M^+^); elemental analysis calcd. for C_24_H_22_N_6_O_2_: C, 67.59; H, 5.20; N, 19.71. Found: C, 67.67; H, 5.12; N, 19.81.

##### 4-((4,6-bis(diethylamino)-1,3,5-triazin-2-yl)amino)benzoic acid (16)

The product was obtained as white solid, *Method A* 0.77 g (85.9%) yield; *Method B* 0.82 g (92%) yield; mp: 280–283 °C; IR (KBr): 3700–2700 (br, OH, acid), 3420 (NH, amine), 1683 (CO, acid) cm^−1^; ^1^H-NMR (500 MHz, DMSO-d_6_): δ 1.09 (des. t, 12H, 4CH_3_), 3.50–3.61 (m, 8H, 4CH_2_), 7.77–7.86 (m, 4H, Ar–H), 9.27 (s, 1H, NH, D_2_O exchangeable), 12.48 (s, 1H, OH, D_2_O exchangeable); ^13^C-NMR (125 MHz, DMSO-d_6_): 13.77, 13.95, 41.22, 118.52, 119.13, 123.54, 145.84, 164.48, 167.67. EIMS (m/z): 358.144 (M^+^); elemental analysis calcd. for C_18_H_26_N_6_O_2_: C, 60.32; H, 7.31; N, 23.45. Found: C, 60.22; H, 7.40; N, 23.52.

##### 4-((4,6-dimorpholino-1,3,5-triazin-2-yl)amino)benzoic acid (17)

The product was obtained as white solid, *Method A* 0.72 g (74.5%) yield; *Method B* 0.85 g (88%) yield; mp: 292–294 °C; IR (KBr): 3700–2500 (br, OH, acid), 3438 (NH, amine), 1711 (CO, acid) cm^−1^; ^1^H-NMR (500 MHz, DMSO-d_6_): δ 3.60–3.69 (m, 16H, 8CH_2_), 7.73 (d, 2H, *J* = 8.4 Hz, Ar–H), 7.83 (d, 2H, *J* = 8.4 Hz, Ar–H), 9.74–9.87 (m, 1H, NH, D_2_O exchangeable). Elemental analysis calcd. for C_18_H_22_N_6_O_4_: C, 55.95; H, 5.74; N, 21.75. Found: C, 56.01; H, 5.63; N, 21.64.

##### 3.1.3.5.4-((4,6-di(piperidin-1-yl)-1,3,5-triazin-2-yl)amino)benzoic acid (18)

The product was obtained as white solid, *Method A* 0.82 g (85.8%) yield; *Method B* 0.89 g (93.1%) yield; mp: 284–286 °C; IR (KBr): 3700–2500 (br, OH, acid), 3291 (NH, amine), 1691 (CO, acid) cm^−1^; ^1^H-NMR (500 MHz, DMSO-d_6_): δ 0.84–0.87 (m, 6H, 3CH_2_-pip), 1.49–1.51 (m, 4H, 2CH_2_-pip), 3.18–3.20 (m, 10H, 5CH_2_-pip), 7.77–7.90 (m, 4H, Ar–H), 9.15–9.55 (m, 1H, NH, D_2_O exchangeable), 12.52 (brs, 1H, OH, D_2_O exchangeable); ^13^C-NMR (125 MHz, DMSO-d_6_): 11.94, 23.06, 42.31, 118.75, 123.16, 130.52, 145.73, 164.21, 165.89, 166.2, 167.84. Elemental analysis calcd. for C_20_H_26_N_6_O_2_: C, 62.81; H, 6.85; N, 21.97. Found: C, 62.73; H, 6.94; N, 22.02.

#### General procedure for the synthesis of 4-((4-substituted-6-morpholino-1,3,5-triazin-2-yl)amino)benzoic acid derivatives (19–22)

##### Method A

Conventional procedure: To a solution of *N*-(4-chloro-6-substituted-1,3,5-triazin-2-yl)aminobenzoic acid (2.5 mmol) and sodium carbonate (0.66 g, 6.25 mmol) in distilled water (15 mL), add a solution of morpholine (0.33 mL, 3.75 mmol) in dioxane (5 mL) with stirring. The reaction mixture was stirred at room temperature for 2 h then refluxed at 70–80 °C for 8–9 h. The reaction mixture was neutralized with 1 N HCl after cooling. The corresponding crude products were filtered, dried, and recrystallized from ethanol.

##### Method B

Microwave-irradiation: Employing a multimode reactor (Synthos 3000, Aton Paar GmbH, 1400 W maximum magnetron); the initial step was conducted with 4-Teflon vessels rotor (MF 100) that allow processing four reactions under the same conditions. Each vessel has *N*-(4-chloro-6-substituted-1,3,5-triazin-2-yl)aminobenzoic acid (2.5 mmol) and sodium carbonate (0.66 g, 6.25 mmol) mixed with morpholine (0.33 mL, 3.75 mmol) in 3 mL dioxane/water (1:1). The individual vessels were purged with nitrogen gas for 5 min and then were placed in the corresponding rotor, fixed by screwing down the upper rotor place, and finally the rotor was closed with a protective hood. The vessels were heated for 5 min at 100 °C and held at the same temperature for a further 5 min (~2 bar pressure, 400 W). Cooling was accomplished by a fan (5 min). The reaction mixture was neutralized with 1 N HCl after cooling. The corresponding crude products were filtered, dried, and recrystallized from ethanol.

##### 4-((4-morpholino-6-(phenylamino)-1,3,5-triazin-2-yl)amino)benzoic acid (19)

The product was obtained as white solid, *Method A* 0.88 g (89.7%) yield; *Method B* 0.90 g (92%) yield; mp: 317–320 (dec.) °C; IR (KBr): 3750–2700 (br, OH, acid), 3410 (NH, amine), 1687 (CO, acid) cm^−1^; ^1^H-NMR (500 MHz, DMSO-d_6_): δ 3.64 (des. t, 4H, 2CH_2_–N), 3.73 (des. t, 4H, 2CH_2_–O), 6.95–7.96 (m, 9H, Ar–H), 9.28–9.34 (m, 1H, NH, D_2_O exchangeable), 9.55–9.59 (m, 1H, NH, D_2_O exchangeable), 12.46 (s, 1H, OH, D_2_O exchangeable); ^13^C-NMR (125 MHz, DMSO-d_6_): 44.01, 66.52, 119.29, 121.02, 122.61,123.94, 128.96, 130.62, 140.40, 145.04, 164.54, 164.61, 165.22, 167.76. Elemental analysis calcd. for C_20_H_20_N_6_O_3_: C, 61.21; H, 5.14; N, 21.42. Found: C, 61.31; H, 5.04; N, 21.32.

##### 4-((4-(benzylamino)-6-morpholino-1,3,5-triazin-2-yl)amino)benzoic acid (20)

The product was obtained as white solid, *Method A* 0.85 g (83.7%) yield; *Method B* 0.92 g (90.6%) yield; mp: 229–232 °C; IR (KBr): 3700–2600 (br, OH, acid), 3410 (NH, amine), 1687 (CO, acid) cm^−1^; ^1^H-NMR (500 MHz, DMSO-d_6_): δ 3.57-3.74 (m, 8H, 4CH_2_), 4.45–4.50 (m, 2H, benzyl CH_2_), 7.18–7.71 (m, 4H, Ar–H), 7.69–7.85 (m, 5H, Ar–H), 9.49–9.73 (m, 1H, NH, D_2_O exchangeable), 12.40 (s, 1H, OH, D_2_O exchangeable); ^13^C-NMR (125 MHz, DMSO-d_6_): 43.93, 44.09, 66.48, 119.00, 123.60, 127.18, 128.73, 130.56, 140.75, 145.12, 164.54, 164.61, 165.22, 167.66. Elemental analysis calcd. for C_21_H_22_N_6_O_3_: C, 62.06; H, 5.46; N, 20.68. Found: C, 62.17; H, 5.58; N, 20.48.

##### 4-((4-(diethylamino)-6-morpholino-1,3,5-triazin-2-yl)amino)benzoic acid (21)

The product was obtained as white solid, *Method A* 0.78 g (83.8%) yield; *Method B* 0.84 g (90.2%) yield; mp: 258–261 °C; IR (KBr): 3700–2500 (br, OH, acid), 3391 (NH, amine), 1687 (CO, acid) cm^−1^; ^1^H-NMR (500 MHz, DMSO-d_6_): δ 1.10 (des. t, 6H, 2 CH_3_), 3.49–3.52 (m, 4H, 2CH_2_), 3.58-3.73 (m, 8H, 4CH_2_), 7.81 (m, 4H, Ar–H), 9.45 (m, 1H, NH, D_2_O exchangeable), 12.52 (s, 1H, OH, D_2_O exchangeable); ^13^C-NMR (125 MHz, DMSO-d_6_): 13.60, 13.77, 41.38, 43.90, 66.52, 118.79, 123.46, 130.66, 145.35, 164.02, 164.69, 164.88, 167.68. Elemental analysis calcd. for C_18_H_24_N_6_O_3_: C, 58.05; H, 6.50; N, 22.57. Found: C, 58.00; H, 6.48; N, 22.52.

##### 4-((4-morpholino-6-(piperidin-1-yl)-1,3,5-triazin-2-yl)amino)benzoic acid (22)

The product was obtained as white solid, *Method A* 0.79 g (82.2%) yield; *Method B* 0.88 g (91.6%) yield; mp: 248–250 °C; IR (KBr): 3700–2700 (br, OH, acid), 3292 (NH, amine), 1656 (CO, acid) cm^−1^; ^1^H-NMR (500 MHz, DMSO-d_6_): δ 0.85–0.88 (m, 3H, pip), 1.50–1.51 (m, 2H, CH_2_-pip), 3.21–3.68 (m, 13H, 4CH_2_-mor, 5H-pip), 7.81–7.85 (m, 4H, Ar–H), 9.54–9.65 (m, 1H, NH, D_2_O exchangeable), 12.41 (brs, 1H, OH, D_2_O exchangeable); ^13^C-NMR (125 MHz, DMSO-d_6_): 12.02, 23.01, 42.64, 44.01, 66.48, 119.09, 123.77, 130.62, 145.87, 164.48, 165.91, 167.65. Elemental analysis calcd. for C_19_H_24_N_6_O_3_: C, 59.36; H, 6.29; N, 21.86. Found: C, 59.31; H, 6.22; N, 21.75.

#### General procedure for the synthesis of methyl 4-((4,6-disubsituted-1,3,5-triazin-2-yl)amino)benzoic acid derivatives (23–31)

Concentrated sulphuric acid (0.5 mL, 99%) was added to a cooled suspension of *N*-(4,6-disubsituted-1,3,5-triazin-2-yl)aminobenzoic acid (1 mmol) in methanol (20 mL). The reaction mixture was refluxed for 8–10 h. The reaction mixture was cooled and poured into sodium bicarbonate solution. The corresponding crude products were filtered, dried, and recrystallized from ethanol (Additional file 1).

##### Methyl 4-((4,6-bis(phenylamino)-1,3,5-triazin-2-yl)amino)benzoate (23)

The product was obtained as white solid, 0.39 g (94.6%) yield; mp: 250–252 °C; IR (KBr): 3400 (NH, amine), 1705 (CO, ester) cm^−1^; ^1^H-NMR (500 MHz, DMSO-d_6_): δ 3.80 (s, 3H, OCH_3_), 7.27–7.85 (m, 14H, Ar–H), 9.37–9.95 (m, 3H, NH, D_2_O exchangeable); ^13^C-NMR (125 MHz, DMSO-d_6_): 52.35, 119.57, 121.16, 122.82, 128.96, 129.34, 130.41, 140.19, 145.35, 164.42, 164.52, 166.56. Elemental analysis calcd. for C_23_H_20_N_6_O_2_: C, 66.98; H, 4.89; N, 20.38. Found: C, 66.83; H, 4.77; N, 20.26.

##### Methyl 4-((4,6-bis(benzylamino)-1,3,5-triazin-2-yl)amino)benzoate (24)

The product was obtained as white solid, 0.44 g (99.8%) yield; mp: 272–274 °C; IR (KBr): 3413 (NH, amine), 1711 (CO, ester) cm^−1^; ^1^H-NMR (500 MHz, DMSO-d_6_): δ 3.76 (s, 3H, OCH_3_), 4.42–4.48 (m, 4H, 2CH_2_), 7.18–7.69 (m, 16H, 14 Ar–H, 2NH), 9.30–9.36 (m, 1H, NH, D_2_O exchangeable); ^13^C-NMR (125 MHz, DMSO-d_6_): 43.93, 52.25, 118.87, 127.05, 127.34, 127.41, 127.62, 128.71, 130.31. 141.01, 166.60. Elemental analysis calcd. for C_25_H_24_N_6_O_2_: C, 68.17; H, 5.49; N, 19.08. Found: C, 68.05; H, 5.37; N, 19.01.

##### Methyl 4-((4,6-bis(diethylamino)-1,3,5-triazin-2-yl)amino)benzoate (25)

The product was obtained as white solid, 0.35 g (94.0%) yield; mp: 190–192 °C; IR (KBr): 3338 (NH, amine), 1718 (CO, ester) cm^−1^; ^1^H-NMR (500 MHz, DMSO-d_6_): δ 1.10 (m, 12H, 4CH_3_), 3.48–3.54 (m, 8H, 4CH_2_), 3.76 (s, 3H, OCH_3_), 7.79–7.88 (m, 4H, Ar–H), 9.33 (s, 1H, NH, D_2_O exchangeable); ^13^C-NMR (125 MHz, DMSO-d_6_): 13.77, 13.95, 41.24, 52.19, 118.60, 121.84, 130.41, 146.24, 164.46, 166.60. Elemental analysis calcd. for C_19_H_28_N_6_O_2_: C, 61.27; H, 7.58; N, 22.56. Found: C, 61.15; H, 7.46; N, 22.45.

##### Methyl 4-((4,6-dimorpholino-1,3,5-triazin-2-yl)amino)benzoate (26)

The product was obtained as white solid, 0.32 g (79.9%) yield; mp: 141–144 °C; IR (KBr): 3411 (NH, amine), 1711 (CO, ester) cm^−1^; ^1^H-NMR (500 MHz, DMSO-d_6_): δ 3.60–3.68 (m, 16H, 8CH_2_), 3.77 (s, 3H, OCH_3_), 7.81–7.85 (m, 4H, Ar–H), 9.54 (s, 1H, NH, D_2_O exchangeable); ^13^C-NMR (125 MHz, DMSO-d_6_): 43.88, 52.27, 66.52, 119.08, 122.38, 130.58, 145.54, 164.50, 165.15, 166.63. Elemental analysis calcd. for C_19_H_24_N_6_O_4_: C, 56.99; H, 6.04; N, 20.99. Found: C, 56.87; H, 5.93; N, 20.88.

##### Methyl 4-((4,6-di(piperidin-1-yl)-1,3,5-triazin-2-yl)amino)benzoate (27)

The product was obtained as white solid, 0.35 g (88.3%) yield; mp: 240-242 °C; IR (KBr): 3335 (NH, amine), 1711 (CO, ester) cm^−1^; ^1^H-NMR (500 MHz, DMSO-d_6_): δ 0.84–0.87 (m, 8H, 4CH_2_), 1.48–1.50 (m, 4H, 2CH_2_), 3.18–3.29 (m, 8H, 4CH_2_), 3.77 (s, 3H, OCH_3_), 7.77–7.93 (m, 4H, Ar–H), 9.14-9.37 (m, 1H, NH, D_2_O exchangeable); ^13^C-NMR (125 MHz, DMSO-d_6_): 11.98, 23.06, 42.29, 52.23, 118.75, 119.31, 130.35, 146.20, 164.46, 166.14, 166.65. Elemental analysis calcd. for C_21_H_28_N_6_O_2_: C, 63.62; H, 7.12; N, 21.20. Found: C, 63.53; H, 7.05; N, 21.09.

##### Methyl 4-((4-morpholino-6-(phenylamino)-1,3,5-triazin-2-yl)amino)benzoate (28)

The product was obtained as white solid, 0.28 g (69.0%) yield; mp: 232–234 °C; IR (KBr): 3404(NH, amine), 1709 (CO, ester) cm^−1^; ^1^H-NMR (500 MHz, DMSO-d_6_): δ 3.64 (des. t, 4H, 2CH_2_-N), 3.73 (des. t, 4H, 2CH_2_-O), 3.77 (s, 3H, OCH_3_), 6.95–7.96 (m, 9H, Ar–H), 9.29–9.35 (m, 1H, NH, D_2_O exchangeable), 9.61–9.71 (m, 1H, NH, D_2_O exchangeable); ^13^C-NMR (125 MHz, DMSO-d_6_): 44.03, 52.31, 66.52, 119.34, 119.53, 120.77, 122.61, 128.96, 130.48, 140.39, 145.48, 164.52, 165.20, 166.59. Elemental analysis calcd. for C_21_H_22_N_6_O_3_: C, 62.06; H, 5.46; N, 20.68. Found: C, 61.97; H, 5.33; N, 20.57.

##### Methyl 4-((4-(benzylamino)-6-morpholino-1,3,5-triazin-2-yl)amino)benzoate (29)

The product was obtained as white solid, 0.34 g (80.9%) yield; mp: 155–158 °C; IR (KBr): 3340 (NH, amine), 1714 (CO, ester) cm^−1^; ^1^H-NMR (500 MHz, DMSO-d_6_): δ 3.60–3.66 (m, 8H, 4CH_2_), 3.77 (s, 3H, OCH_3_), 4.45–4.50 (dd, 2H, *J* = 23.7, 6.1 Hz, benzyl CH_2_), 7.17–7.30 (m, 4H, Ar–H), 7.63–7.88 (m, 5H, Ar–H), 9.42–9.47 (m, 1H, NH, D_2_O exchangeable); ^13^C-NMR (125 MHz, DMSO-d_6_): 43.84, 44.07, 52.25, 66.50, 79.63, 118.98, 122.24, 127.15, 127.34, 127.95, 128.71, 130.45, 140.88, 145.84, 164.57, 165.18, 166.48, 166.62. EIMS (m/z): 420.036 (M^+^); elemental analysis calcd. for C_22_H_24_N_6_O_3_: C, 62.84; H, 5.75; N, 19.99. Found: C, 62.76; H, 5.65; N, 19.87.

##### Methyl 4-((4-(diethylamino)-6-morpholino-1,3,5-triazin-2-yl)amino)benzoate (30)

The product was obtained as white solid, 0.28 g (72.5%) yield; mp: 151–153 °C; IR (KBr): 3337 (NH, amine), 1717 (CO, ester) cm^−1^; ^1^H-NMR (500 MHz, DMSO-d_6_): δ 1.10 (des. t, 6H, 2 CH_3_), 3.49–3.54 (m, 4H, 2CH_2_), 3.59–3.68 (m, 8H, 4CH_2_), 3.77 (s, 3H, OCH_3_), 7.82–7.86 (m, 4H, Ar–H), 9.44 (s, 1H, NH, D_2_O exchangeable); ^13^C-NMR (125 MHz, DMSO-d_6_): 13.62, 13.77, 41.30, 43.86, 66.54, 79.70, 118.81, 122.11, 130.48, 145.92, 164.50, 165.22, 166.58. Elemental analysis calcd. for C_19_H_26_N_6_O_3_: C, 59.05; H, 6.78; N, 21.75. Found: C, 59.01; H, 6.67; N, 21.65.

##### Methyl 4-((4-morpholino-6-(piperidin-1-yl)-1,3,5-triazin-2-yl)amino)benzoate (31)

The product was obtained as white solid, 0.26 g (65.3%) yield; mp: 140–142 °C; IR (KBr): 3342 (NH, amine), 1714 (CO, ester) cm^−1^; ^1^H-NMR (500 MHz, DMSO-d_6_): δ 1.47–1.59 (m, 6H, 3CH_2_, pip), 3.54-3.69 (m, 12H, 4CH_2_ mor, 2CH_2_ pip), 3.77 (s, 3H, OCH_3_), 7.80-7.82 (m, 4H, Ar–H), 9.46 (s, 1H, NH, D_2_O exchangeable); ^13^C-NMR (125 MHz, DMSO-d_6_): 24.86, 25.94, 43.91, 44.22, 52.23, 66.56, 118.94, 122.21, 130.54, 145.78, 164.57, 164.73, 165.36, 166.58. Elemental analysis calcd. for C_20_H_26_N_6_O_3_: C, 60.29; H, 6.58; N, 21.09. Found: C, 60.20; H, 6.49; N, 21.02.

### Biology

The microdilution susceptibility test in Müller-Hinton Broth (Oxoid) and Sabouraud Liquid Medium (Oxoid) were used for the determination of antibacterial and antifungal activity [45]. The utilized test organisms were: *Escherichia coli* (*E. coli*) ATCC 25922 as an example of Gram-negative bacteria*, Staphylococcus aureus* (*S. aureus*) ATCC 19433 as an example of Gram-positive bacteria and *Candida albicans* (*C. albicans*) as yeast-like fungi. Ampicillin trihydrate and clotrimazole were used as standard antibacterial and antifungal agents, respectively. Solutions of the test compounds, ampicillin trihydrate and clotrimazole were prepared in DMSO to a concentration of 1600 μg/mL. Twofold dilutions of the compounds were prepared ($800, 400, \ldots 6.25$ μg/mL). Microorganism suspensions at 106 CFU/mL (Colony Forming Unit/mL) concentrations were inoculated to the corresponding wells. Plates were incubated at 36 °C for 24–48 h. The incubation chamber was kept sufficiently humid. At the end of the incubation period, the minimal inhibitory concentrations (MIC) were determined.

Invitro cytotoxicity of the test compounds (**10**) and (**13**) were carried out with 5ero cell line using Mosmann method with certain modifications as described in the literature [46]. Briefly the cells were incubated for 72 h with different dilutions of selected compounds using MTT as reagent for the detection of cytotoxicity. Results were expressed as half maximal cytotoxic concentration (CC_50_) of the fibroblast cells in µg/mL. 50% cytotoxic concentration (CC_50_) values represent the concentration of compound required to kill 50% of the fibroblast cells. The selectivity index (SI) was calculated using the formula, SI = CC_50_/MIC.

## Conclusions

Synthesis and characterization of mono-, di- and trisubstituted s-triazine derivatives, containing 4-aminobenzoic acid moiety were described. The 1,3,5-triazine tripod series were prepared by conventional method or by using microwave irradiation, employing a multimode reactor (Synthos 3000, Aton Paar GmbH, 1400 W maximum magnetron). Using microwave irradiation gave the desired products in less time, good yield and higher purity. Esterification of the 4-aminobenzoic acid moiety afforded methyl ester analogues. All synthesized compounds were evaluated for their antimicrobial activity. Where, some of which showed either comparable or 50% activity of that of ampicillin against S. aureus and E. coli. All compounds had lower antifungal activity than clotrimazole (Canesten^®^, Bayer). Compounds (**10**), (**16**), (**25**) and (**30**) have antimicrobial activity against S. aureus comparable to that of ampicillin, while the activity of compound (**13**) is about 50% of that of ampicillin. Compounds (**13**) and (**14**) have antimicrobial activity against E. coli comparable to that of ampicillin, while the activity of compounds (**9**–**12**) and (**15**) is about 50% of that of ampicillin.

Furthermore, minimum inhibitory concentrations values for clinical isolates of compounds (**10**), (**13**), (**14**), (**16**)**, (25**) and (**30**) were tested. Compounds (**10**) and (**13**) were more active against *MRSA* and *E. coli* than ampicillin. Invitro cytotoxicity results revealed that compounds (**10**) and (**13**) were nontoxic up to 250 µg/mL (with SI = 10) and 125 µg/mL (with SI = 5), respectively. These results prompted us to further pursue in SAR as our future plane.

## Additional file

**Additional file 1.** Additional figures.
