# Supplementary material for: Synthesis, characterization and evaluation of 1,3,5-triazine aminobenzoic acid derivatives for their antimicrobial activity
Source: Chem Cent J. 2017 May 10;11:39. doi: 10.1186/s13065-017-0267-3 (PMC5423881; doi:10.1186/s13065-017-0267-3)
Supplement: Supplementary file 1 — Additional file 1. Additional figures. [file 13065_2017_267_MOESM1_ESM.pdf]

Supplementary information

Synthesis, Characterization and Evaluation of 1,3,5-Triazine Aminobenzoic Acid Derivatives for Their Antimicrobial Activity

Khadijah M. Al-Zaydi[1,*], Hosam H. Khalil[2], Ayman El-Faham[2], Sherine N. Khattab[2,*]

[1] Department of Chemistry, Faculty of Sciences - AL Faisaliah, King Abdulaziz University, Jeddah, P.O. Box 50918, Jeddah 21533, Kingdom Saudi Arabia; E-Mail: kalzaydi@kau.edu.sa (Kh.M.A)

[2] Department of Chemistry, Faculty of Science, Alexandria University, P.O. Box 426, Ibrahimia, Alexandria 21321, Egypt; E-Mail: sh.n.khattab@gmail.com (Sh.N.K); E-mail: aymanel_faham@hotmail.com (A.E), chemhosam1@yahoo.com (H.H.K)

***** Author to whom correspondence should be addressed; E-Mail: kalzaydi@kau.edu.sa(Kh.M.A); sh.n.khattab@gmail.com (Sh.N.K)

Figure number	Figure name	page
1	^{1}H-NMR (500 MHz, DMSO-d$_6$) spectra of 4-((4,6-dichloro-1,3,5-triazin-2yl)amino)benzoic acid **3**	3
2	^{1}H-NMR (500 MHz, DMSO-d$_6$) spectra of *N*-benzyl-4,6-dichloro-1,3,5-triazin-2-amine **5**	4
3	^{1}H-NMR (500 MHz, DMSO-d$_6$) spectra of 4,6-dichlro-*N*,*N*-diethyl-1,3,5-triazin-2-amine **6**	5
4	^{1}H-NMR (500 MHz, DMSO-d$_6$) spectra of 4-(4,6-dichloro-1,3,5-triazin-2-yl)morpholine **7**	6
5	^{1}H-NMR (500 MHz, DMSO-d$_6$) spectra of 2,4-dichloro-6-(piperidin-1-yl)-1,3,5-triazine **8**	7
6	^{1}H- and ^{13}C-NMR (500 MHz, DMSO-d$_6$) spectra of 4-((4-chloro-6-(phenylamino)-1,3,5-triazin-2-yl)amino)benzoic acid **9**	8
7	^{1}H- and^{13}C-NMR (500 MHz, DMSO-d$_6$) spectra of 4-((4-(benzylamino)-6-chloro-1,3,5-triazin-2-yl)amino)benzoic acid **10**	9
8	^{1}H- and ^{13}C-NMR (500 MHz, DMSO-d$_6$) spectra of 4-((4-chloro-6-(diethylamino)-1,3,5-triazin-2-yl)amino)benzoic acid **11**	10

9 ^{1}H- and ^{13}C-NMR (500 MHz, DMSO-d_6) spectra of 4-((4-chloro-6-morpholino-1,3,5-triazin-2-yl)amino)benzoic acid **12** 11
10 ^{1}H-NMR (500 MHz, DMSO-d_6) spectrum of 4-((4-chloro-6-(piperidin-1-yl)-1,3,5-triazin-2-yl)amino)benzoic acid **13** 12
11 ^{1}H- and ^{13}C-NMR (500 MHz, DMSO-d_6) spectra of 4-((4,6-bis(phenylamino)-1,3,5-triazin-2-yl)amino)benzoic acid **14** 13
12 ^{1}H- and ^{13}C-NMR (500 MHz, DMSO-d_6) spectra of 4-((4,6-bis(benzylamino)-1,3,5-triazin-2-yl)amino)benzoic acid **15** 14
13 ^{1}H- and ^{13}C-NMR (500 MHz, DMSO-d_6) spectra of 4-((4,6-bis(diethylamino)-1,3,5-triazin-2-yl)amino)benzoic acid **16** 15
14 ^{1}H-NMR (500 MHz, DMSO-d_6) spectrum of 4-((4,6-dimorpholino-1,3,5-triazin-2-yl)amino)benzoic acid **17** 16
15 ^{1}H- and ^{13}C-NMR (500 MHz, DMSO-d_6) spectra of 4-((4,6-di(piperidin-1-yl)-1,3,5-triazin-2-yl)amino)benzoic acid **18** 17
16 ^{1}H-and ^{13}C-NMR (500 MHz, DMSO-d_6) spectra of 4-((4-morpholino-6-(phenylamino)-1,3,5-triazin-2-yl)amino)benzoic acid **19** 18
17 ^{1}H-and ^{13}C-NMR (500 MHz, DMSO-d_6) spectra of 4-((4-(benzylamino)-6-morpholino-1,3,5-triazin-2-yl)amino)benzoic acid **20** 19
18 ^{1}H-and ^{13}C-NMR (500 MHz, DMSO-d_6) spectra of 4-((4-(diethylamino)-6-morpholino-1,3,5-triazin-2-yl)amino)benzoic acid **21** 20
19 ^{1}H-and ^{13}C-NMR (500 MHz, DMSO-d_6) spectra of 4-((4-morpholino-6-(piperidin-1-yl)-1,3,5-triazin-2-yl)amino)benzoic acid **22** 21
20 ^{1}H-and ^{13}C-NMR (500 MHz, $CDCl_3$) spectra of Methyl 4-((4,6-bis(phenylamino)-1,3,5-triazin-2-yl)amino)benzoate **23** 22
21 ^{1}H-and ^{13}C-NMR (500 MHz, $CDCl_3$) spectra of Methyl 4-((4,6-bis(benzylamino)-1,3,5-triazin-2-yl)amino)benzoate **24** 23
22 ^{1}H-and ^{13}C-NMR (500 MHz, $CDCl_3$) spectra of Methyl 4-((4,6-bis(diethylamino)-1,3,5-triazin-2-yl)amino)benzoate **25** 24
23 ^{1}H-and ^{13}C-NMR (500 MHz, $CDCl_3$) spectra of Methyl 4-((4,6-dimorpholino-1,3,5-triazin-2-yl)amino)benzoate **26** 25
24 ^{1}H-and ^{13}C-NMR (500 MHz, $CDCl_3$) spectra of Methyl 4-((4,6-di(piperidin-1-yl)-1,3,5-triazin-2-yl)amino)benzoate **27** 26
25 ^{1}H-and ^{13}C-NMR (500 MHz, $CDCl_3$) spectra of Methyl 4-((4-morpholino-6-(phenylamino)-1,3,5-triazin-2-yl)amino)benzoate **28** 27
26 ^{1}H-and ^{13}C-NMR (500 MHz, $CDCl_3$) spectra of Methyl 4-((4-(benzylamino)-6-morpholino-1,3,5-triazin-2-yl)amino)benzoate **29** 28
27 ^{1}H-and ^{13}C-NMR (500 MHz, $CDCl_3$) spectra of Methyl 4-((4-(diethylamino)-6-morpholino-1,3,5-triazin-2-yl)amino)benzoate **30** 29
28 ^{1}H-and^{13}C-NMR (500 MHz, $CDCl_3$) spectra of Methyl 4-((4-morpholino-6-(piperidin-1-yl)-1,3,5-triazin-2-yl)amino)benzoate **31** 30

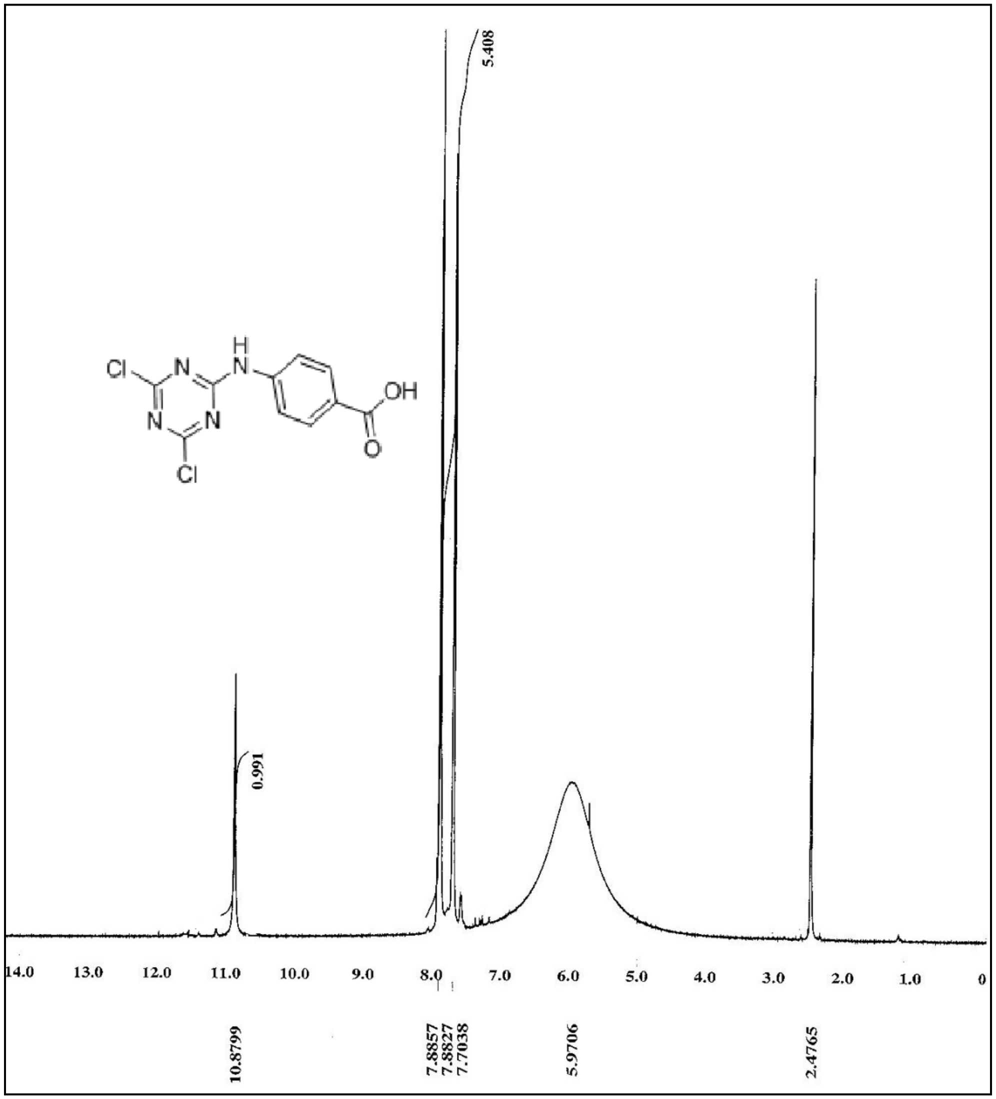

Figure 1: ^{1}H-NMR (500 MHz, DMSO-d_6) spectra of 4-((4,6-dichloro-1,3,5-triazin-2yl)amino)benzoic acid **3**

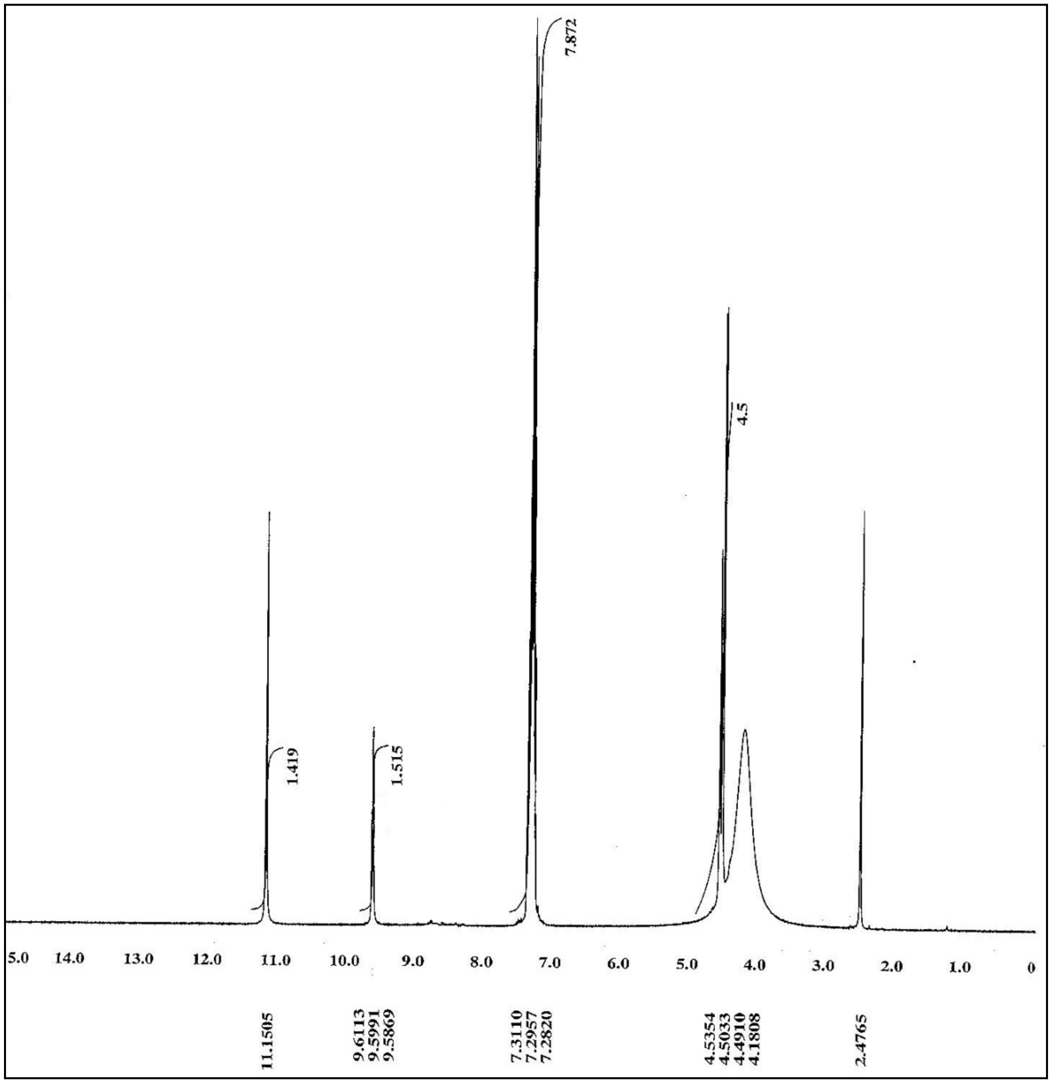

Figure 2: ^{1}H-NMR (500 MHz, DMSO-d_6) spectra of *N*-benzyl-4,6-dichloro-1,3,5-triazin-2-amine **5**

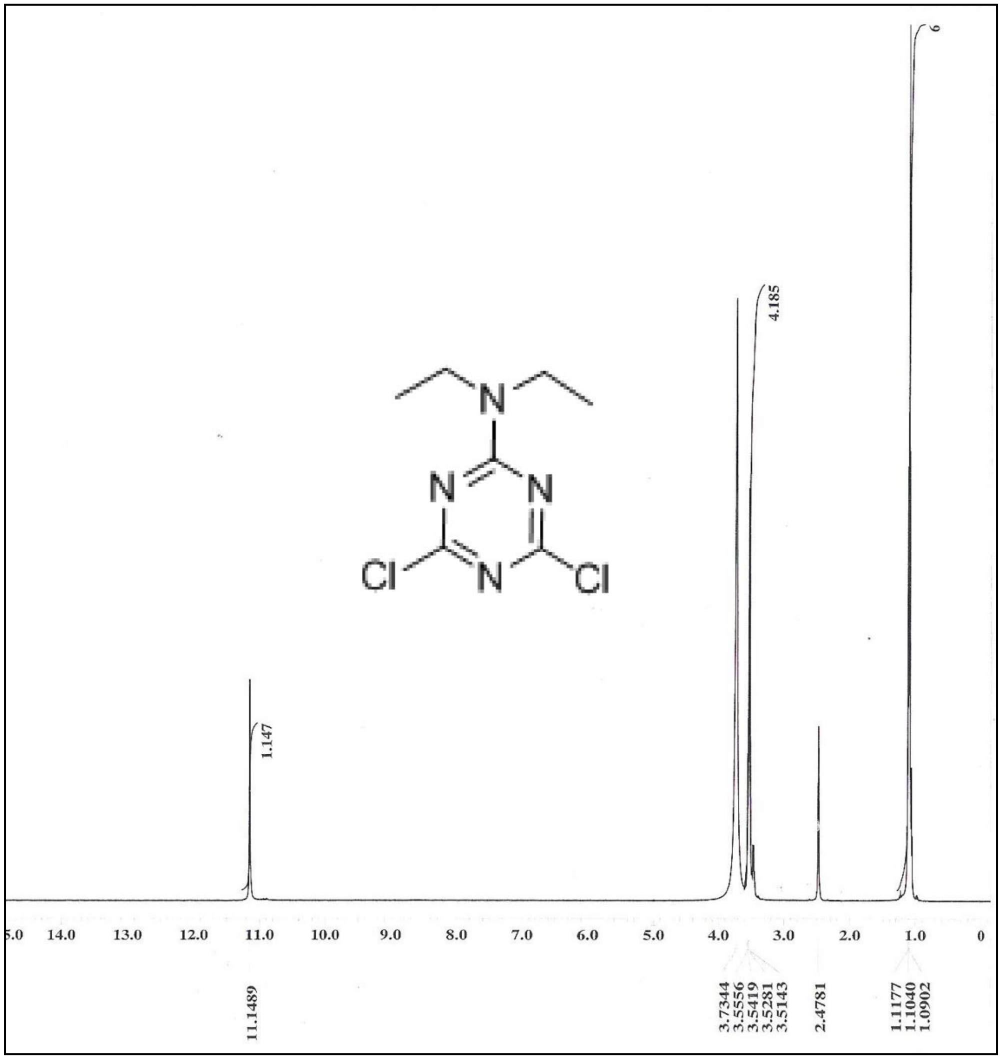

Figure 3: ^{1}H-NMR (500 MHz, DMSO-d_6) spectra of 4,6-dichlro-*N*,*N*-diethyl-1,3,5-triazin-2-amine **6**

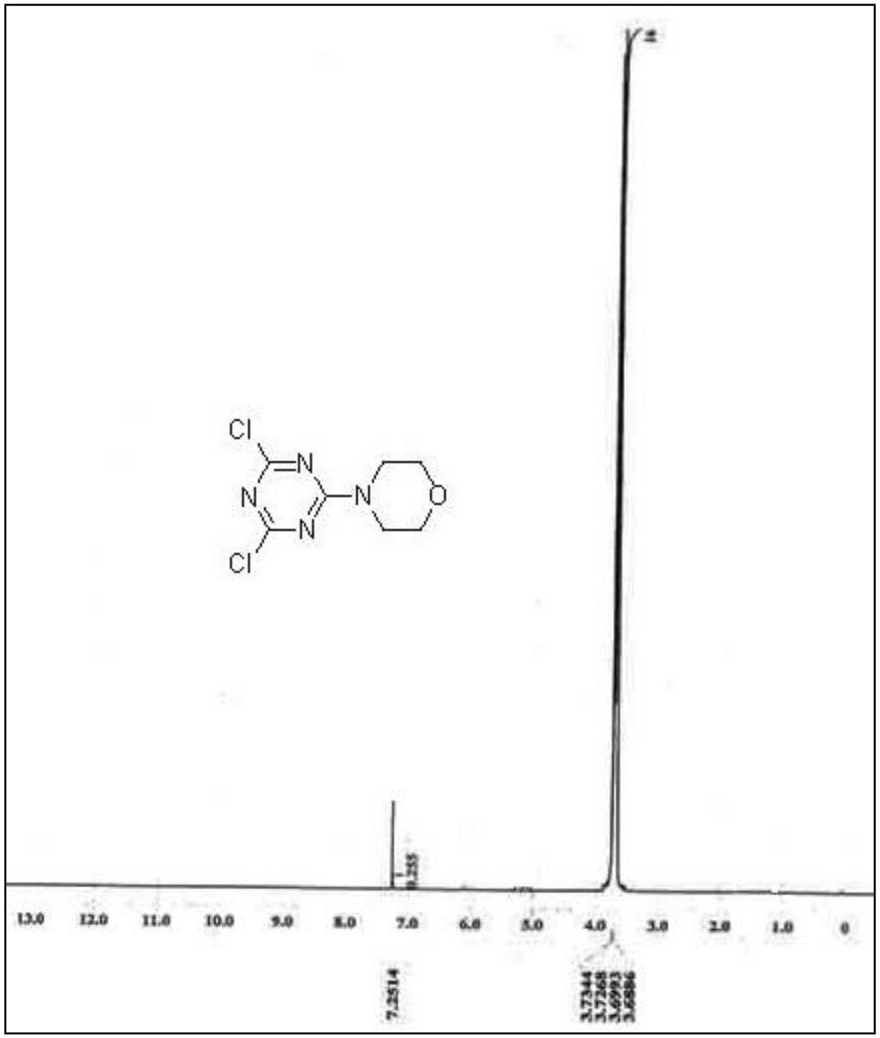

Figure 4: ^{1}H-NMR (500 MHz, DMSO-d_6) spectra of 4-(4,6-dichloro-1,3,5-triazin-2-yl)morpholine **7**

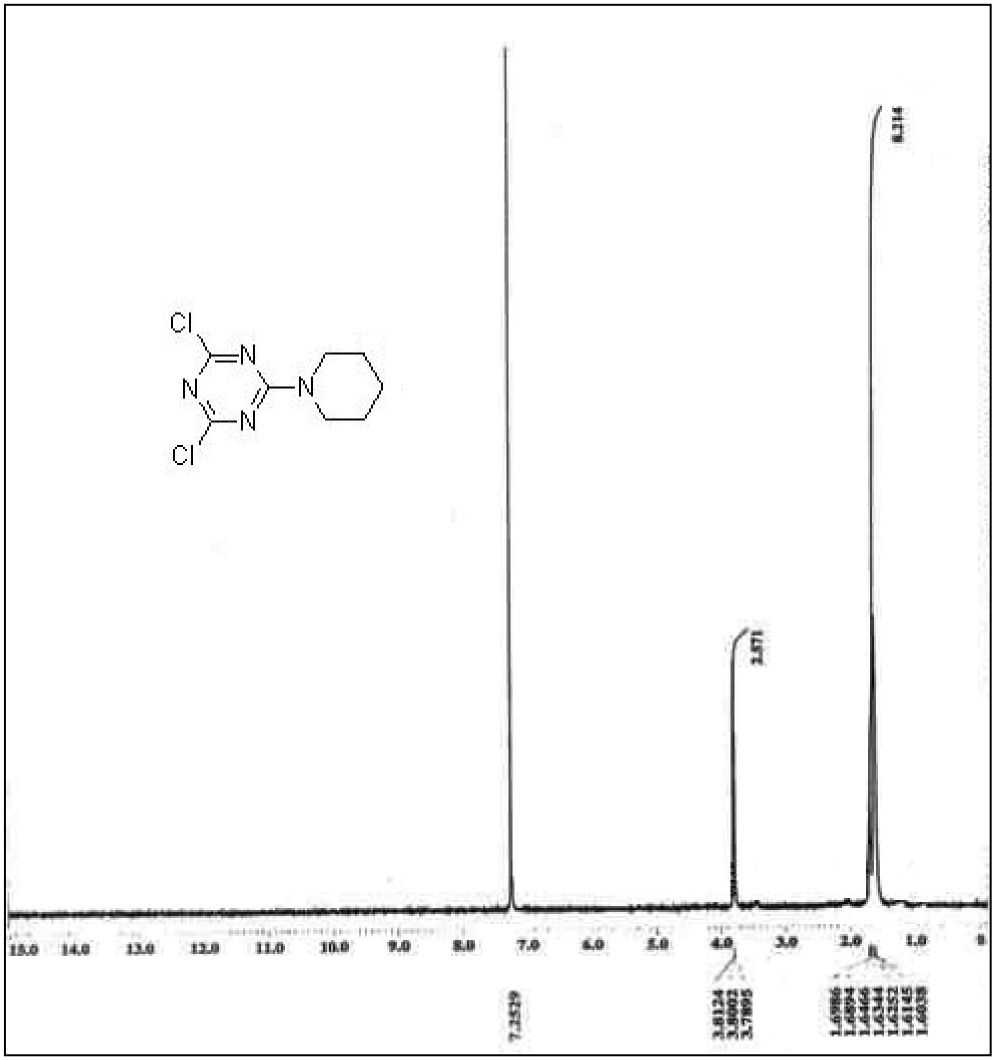

Figure 5: ^{1}H-NMR (500 MHz, DMSO-d_6) spectra of 2,4-dichloro-6-(piperidin-1-yl)-1,3,5-triazine **8**

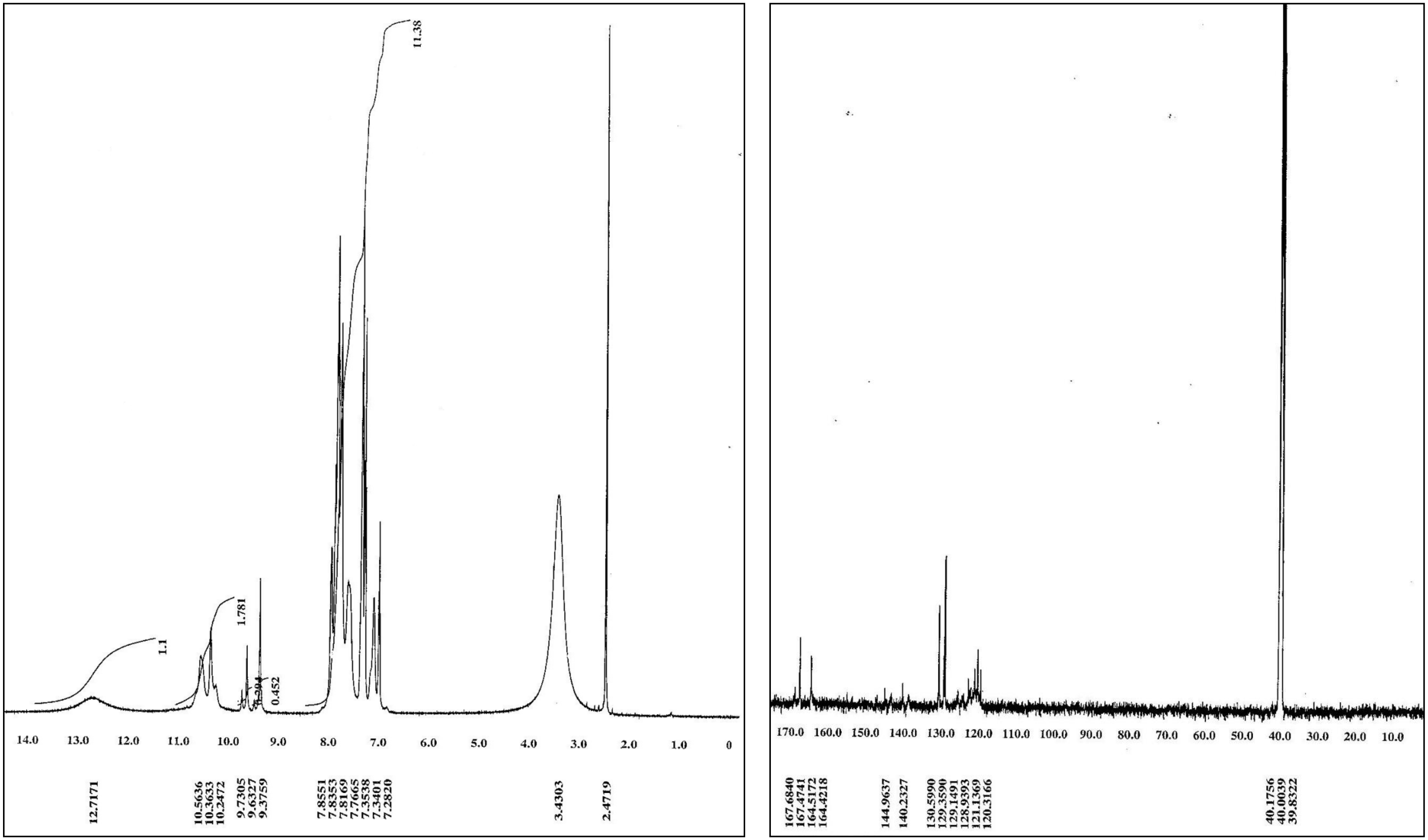

Figure 6: ^{1}H - and ^{13}C-NMR (500 MHz, DMSO-d_6) spectra of 4-((4-chloro-6-(phenylamino)-1,3,5-triazin-2-yl)amino)benzoic acid **9.**

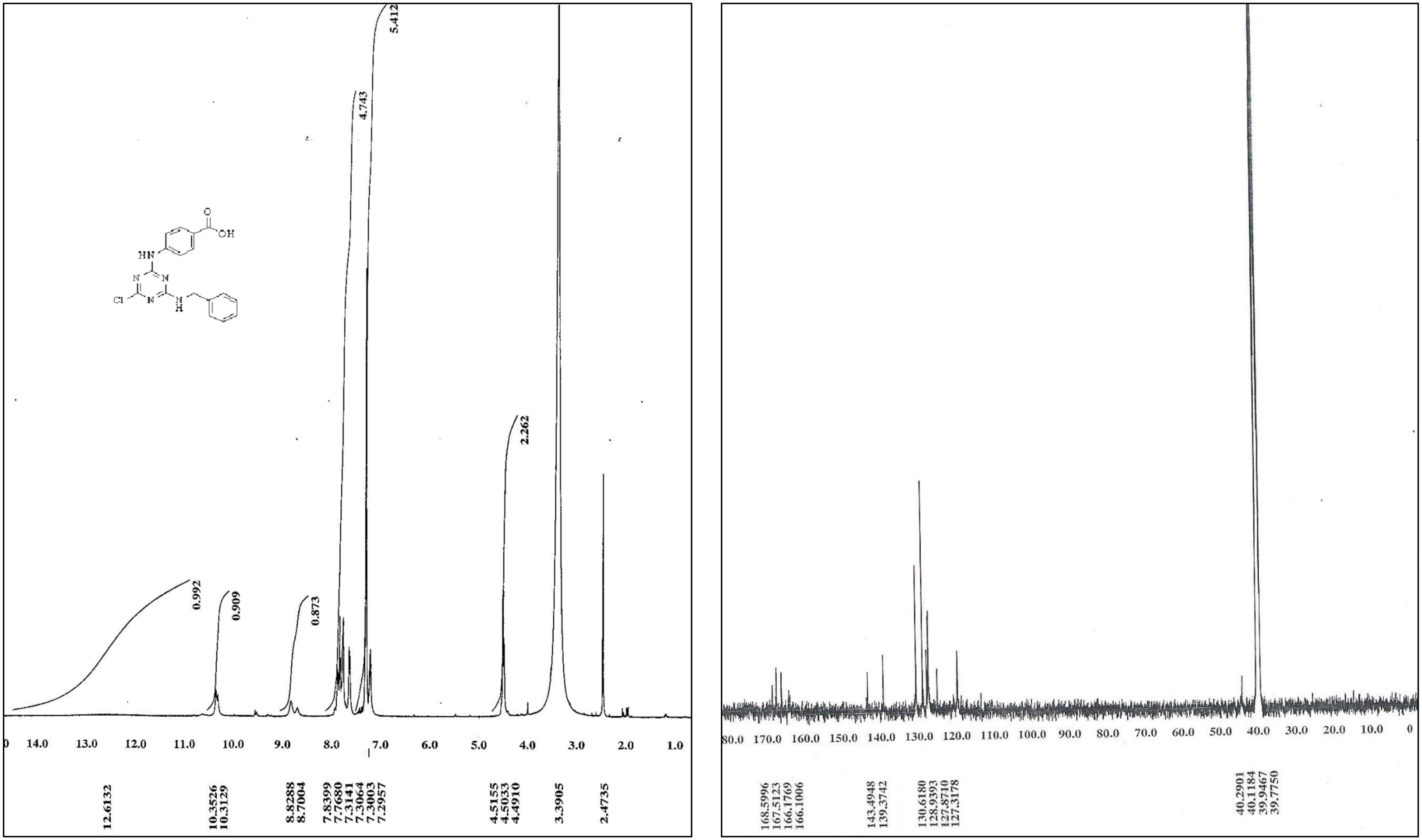

Figure 7:^{1}H- and ^{13}C-NMR (500 MHz, DMSO-d$_6$) spectra of 4-((4-(benzylamino)-6-chloro-1,3,5-triazin-2-yl)amino)benzoic acid **10.**

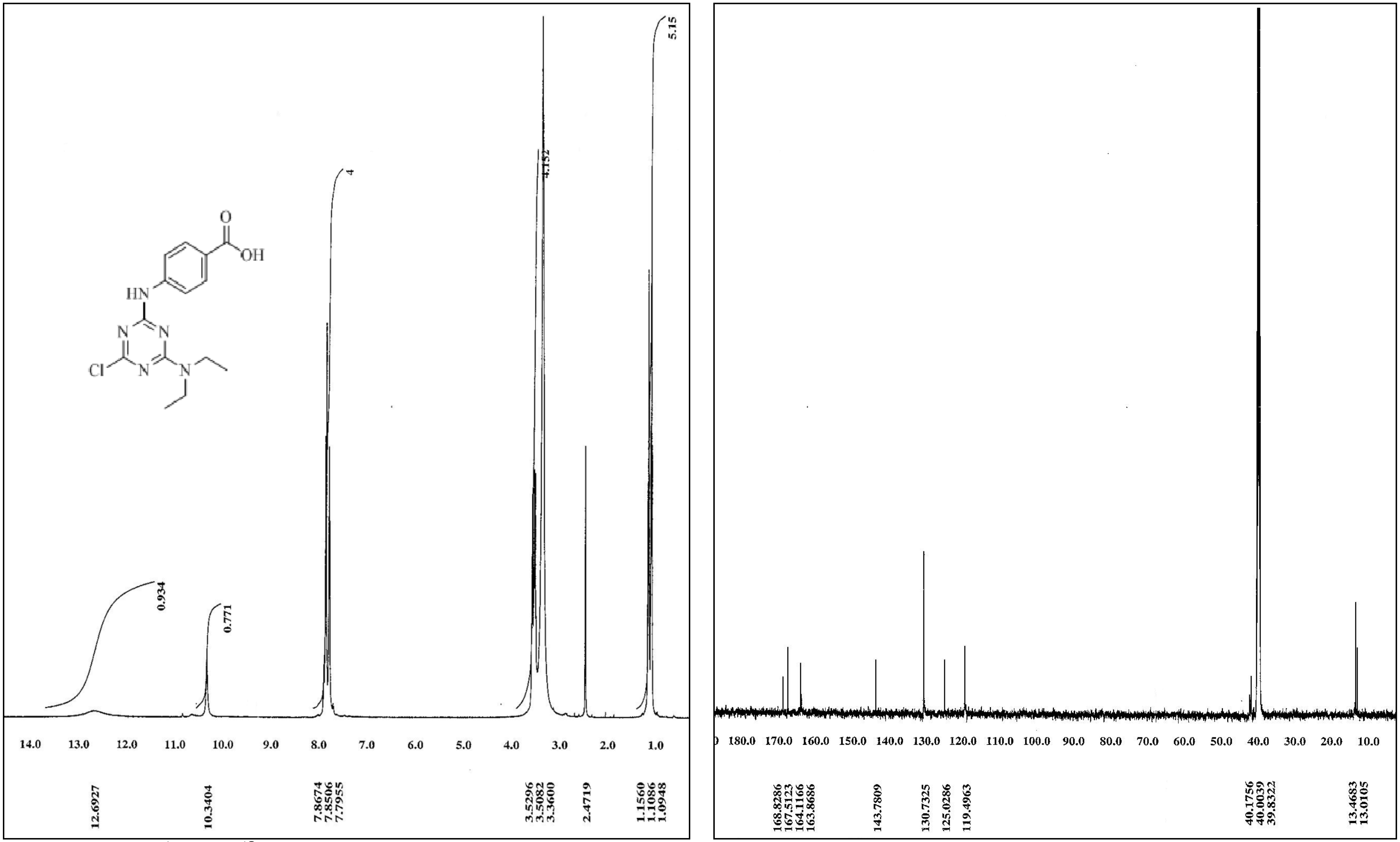

Figure 8: ^{1}H- and ^{13}C-NMR (500 MHz, DMSO-d_6) spectra of 4-((4-chloro-6-(diethylamino)-1,3,5-triazin-2-yl)amino)benzoic acid **11.**

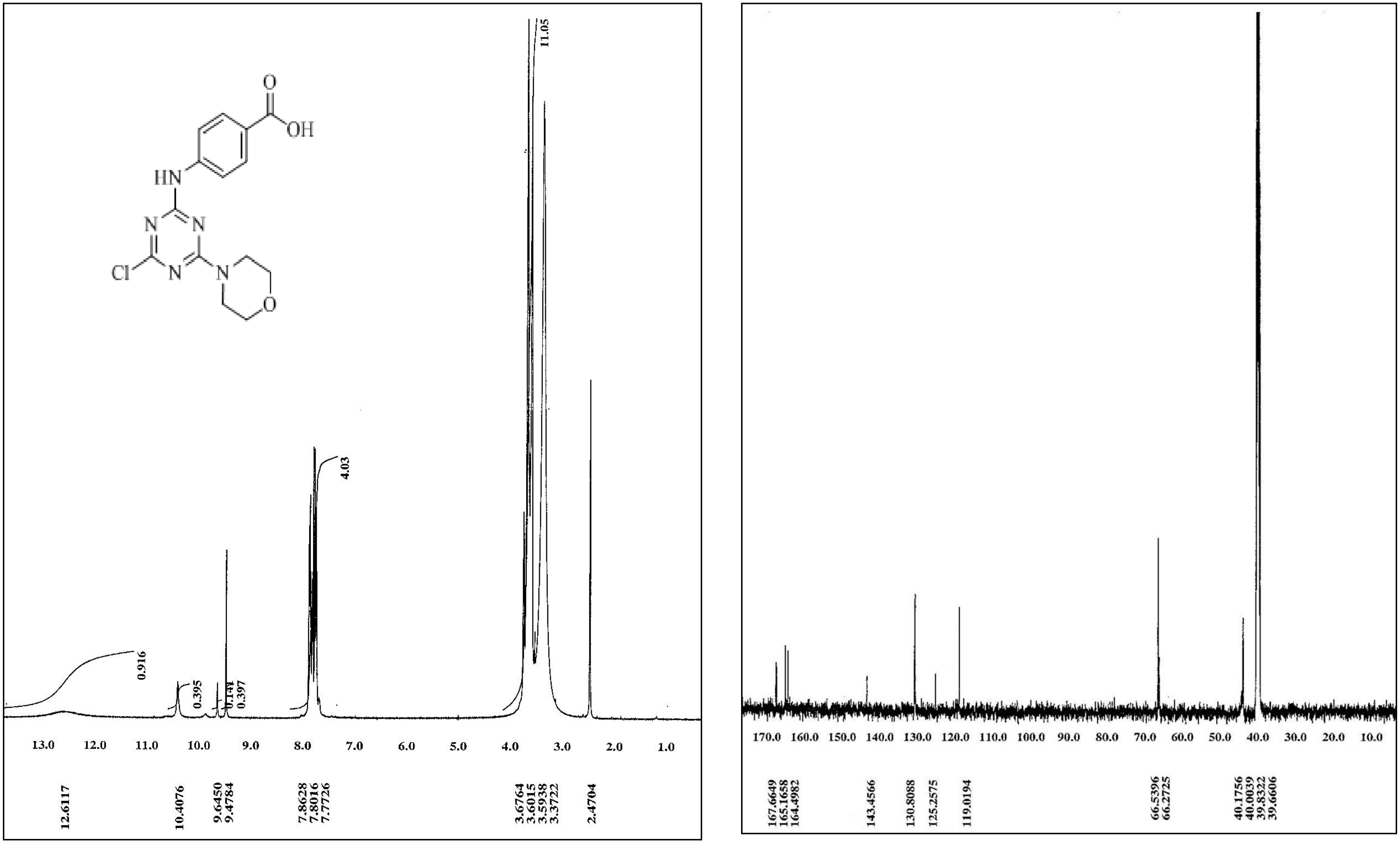

Figure 9: ^{1}H- and ^{13}C-NMR (500 MHz, DMSO-d_6) spectra of 4-((4-chloro-6-morpholino-1,3,5-triazin-2-yl)amino)benzoic acid **12**.

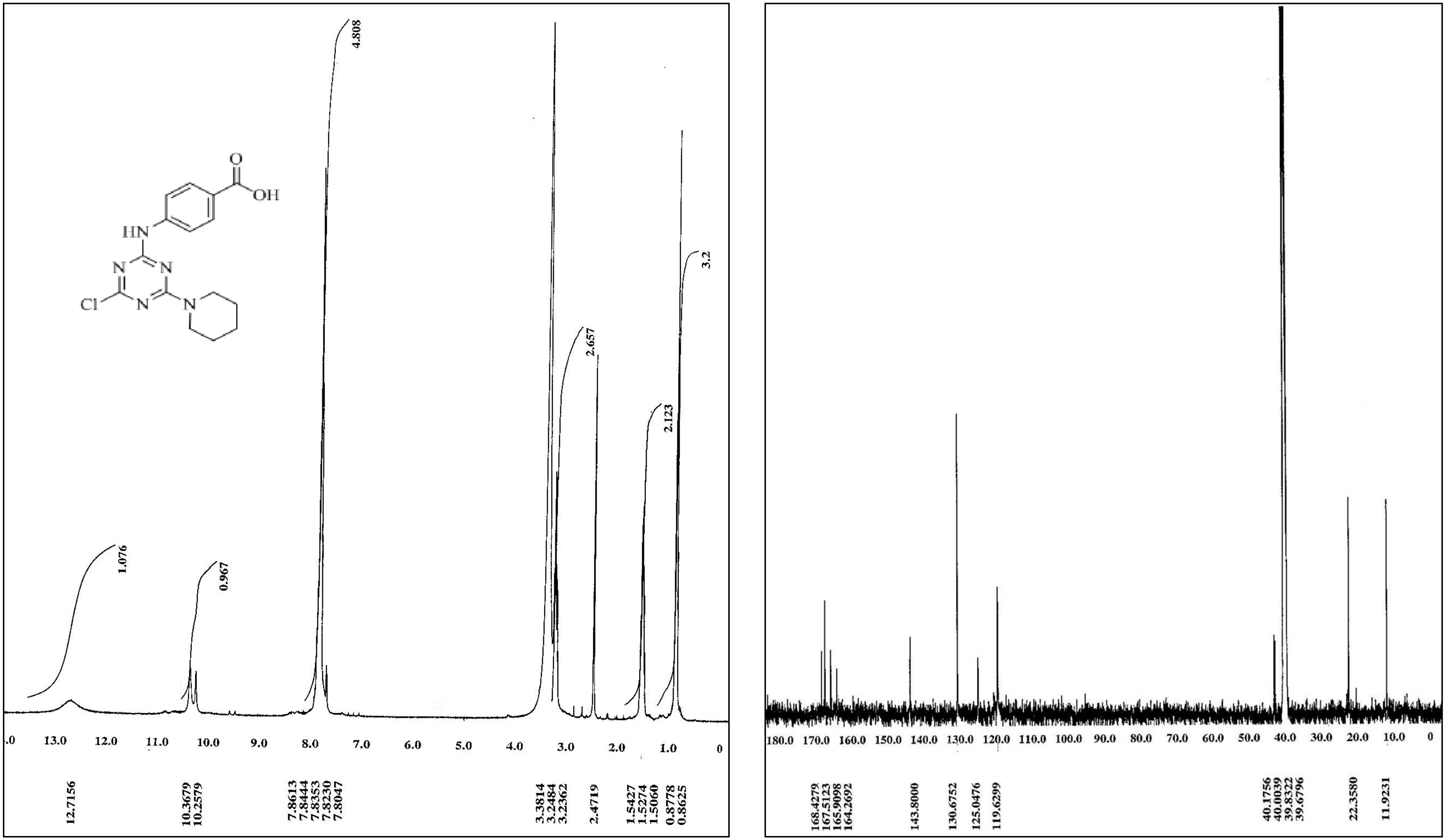

Figure 10: ^{1}H- and ^{13}C-NMR (500 MHz, DMSO-d_6) spectra of 4-((4-chloro-6-(piperidin-1-yl)-1,3,5-triazin-2-yl)amino)benzoic acid **13.**

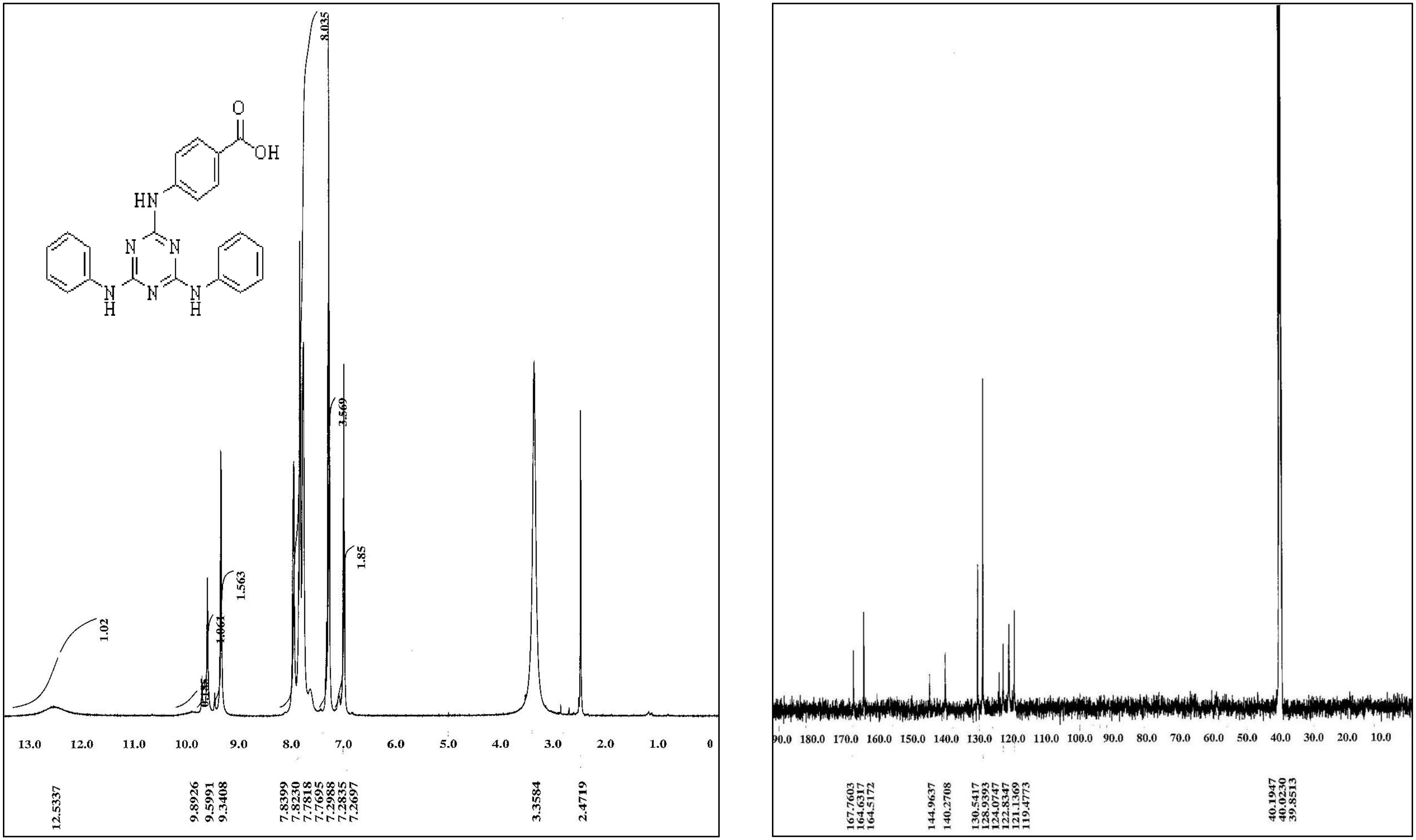

Figure 11: ^{1}H- and ^{13}C-NMR (500 MHz, DMSO-d_6) spectra of 4-((4,6-bis(phenylamino)-1,3,5-triazin-2-yl)amino)benzoic acid **14.**

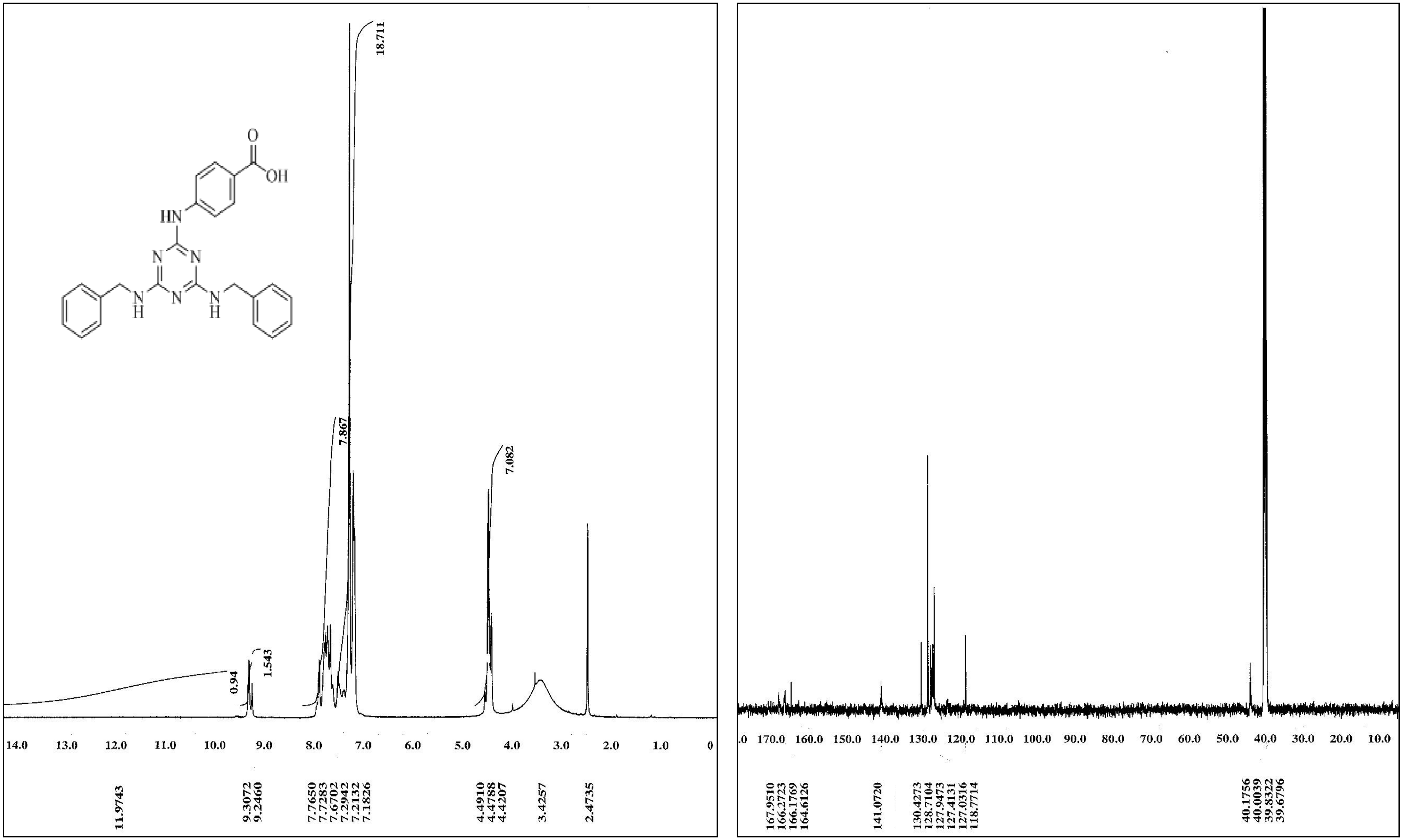

Figure 12:^{1}H- and ^{13}C-NMR (500 MHz, DMSO-d$_6$) spectra of 4-((4,6-bis(benzylamino)-1,3,5-triazin-2-yl)amino)benzoic acid **15.**

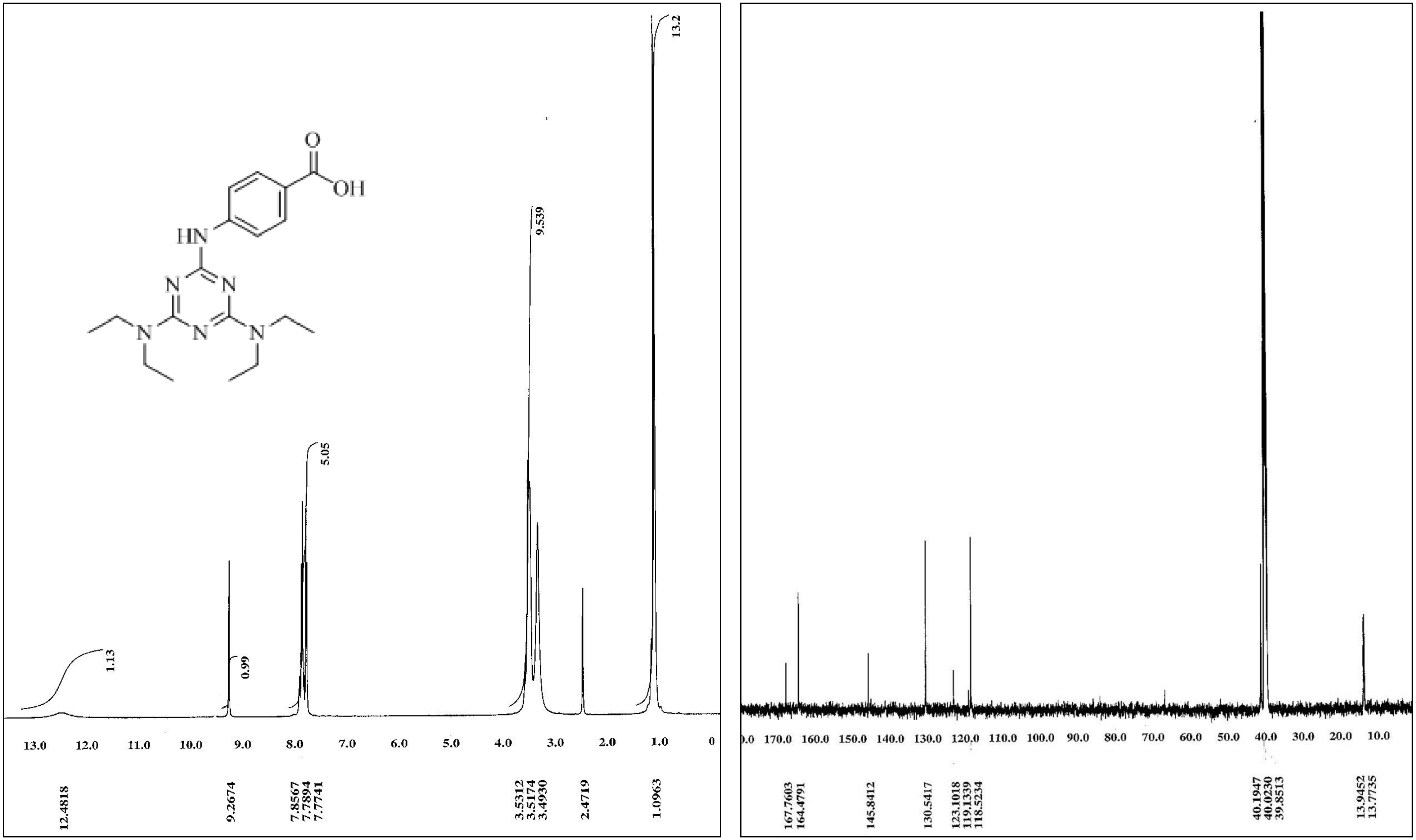

Figure 13: ^{1}H- and ^{13}C-NMR (500 MHz, DMSO-d_6) spectra of 4-((4,6-bis(diethylamino)-1,3,5-triazin-2-yl)amino)benzoic acid **16.**

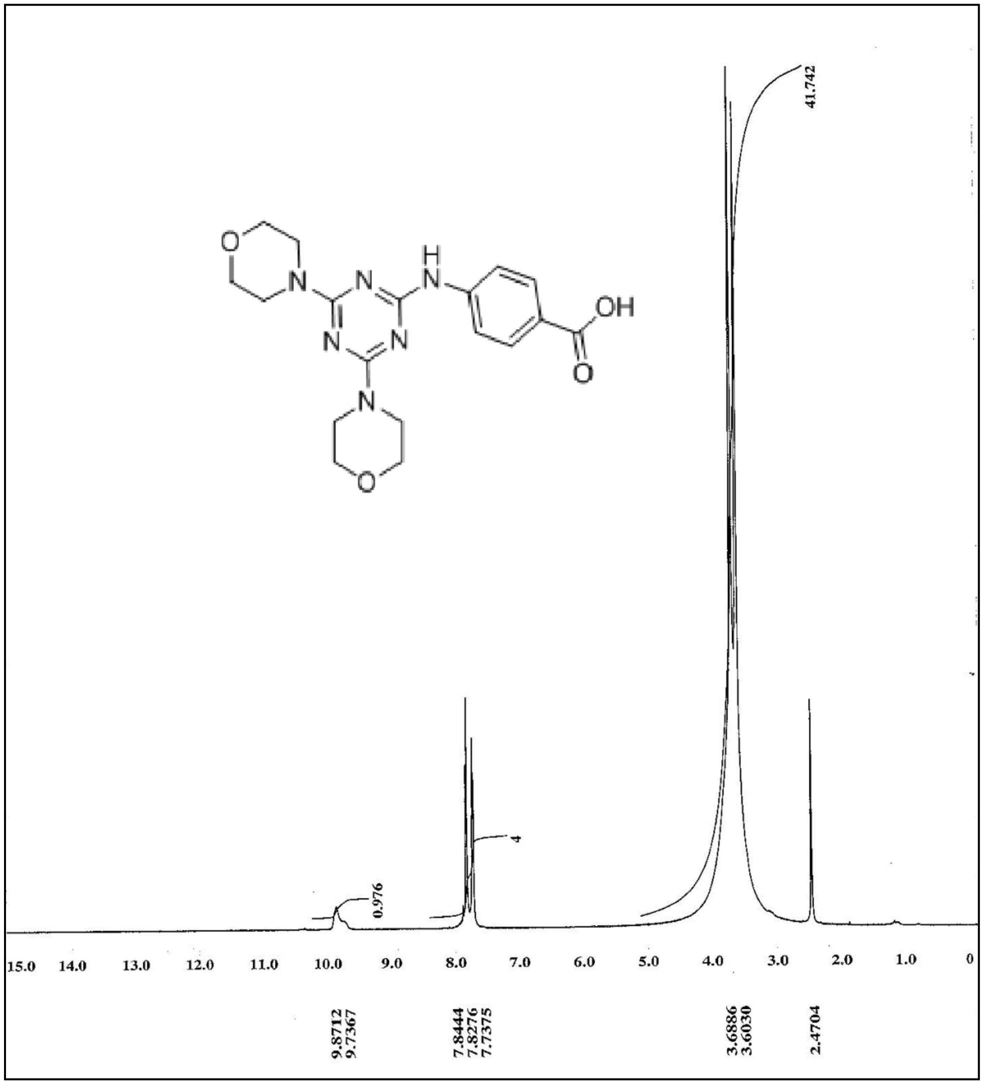

Figure 14:^{1}H-NMR (500 MHz, DMSO-d_6) spectra of 4-((4,6-dimorpholino-1,3,5-triazin-2-yl)amino)benzoic acid **17.**

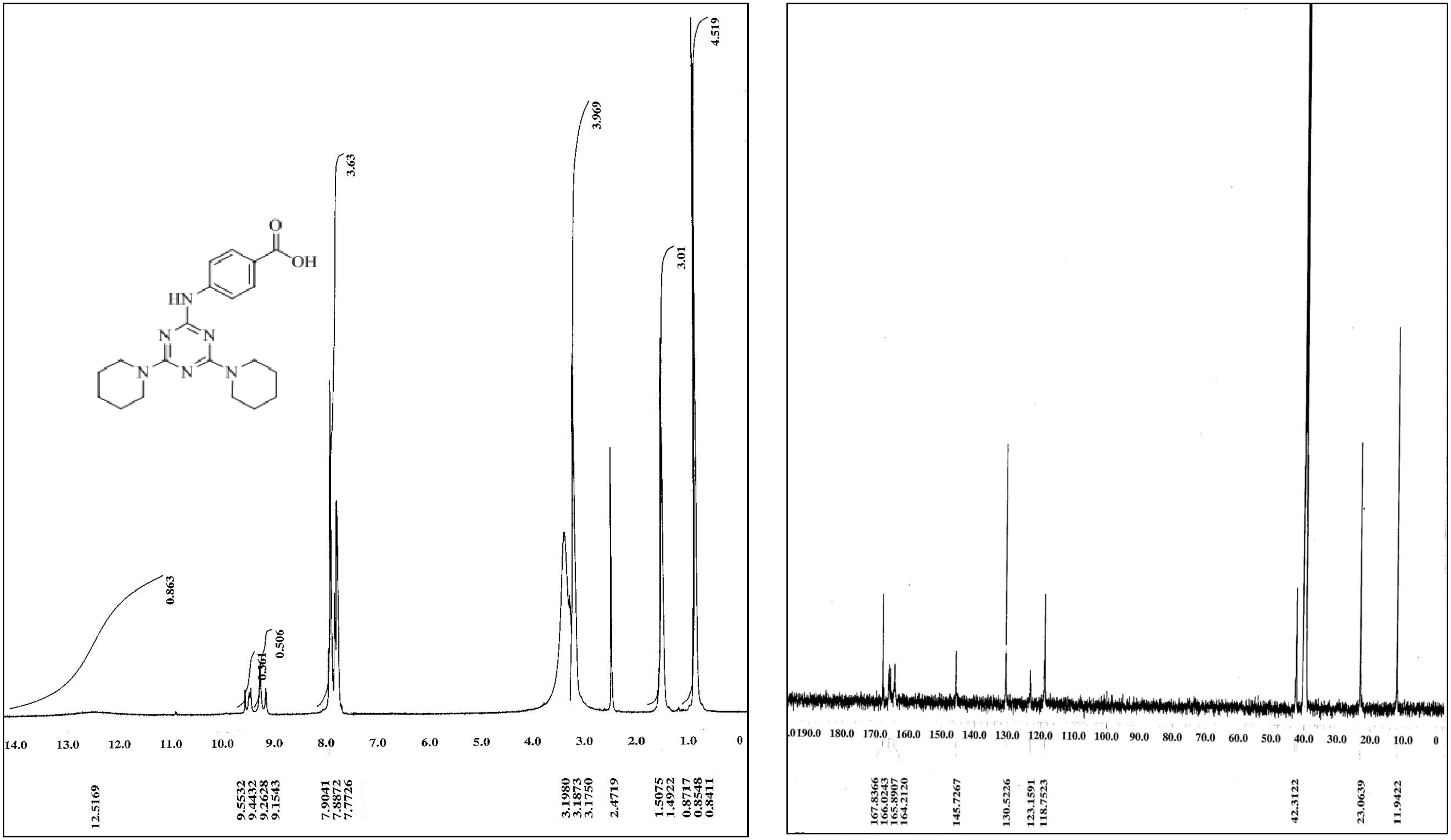

Figure 15:^{1}H- and ^{13}C-NMR (500 MHz, DMSO-d$_6$) spectra of 4-((4,6-di(piperidin-1-yl)-1,3,5-triazin-2-yl)amino)benzoic acid **18.**

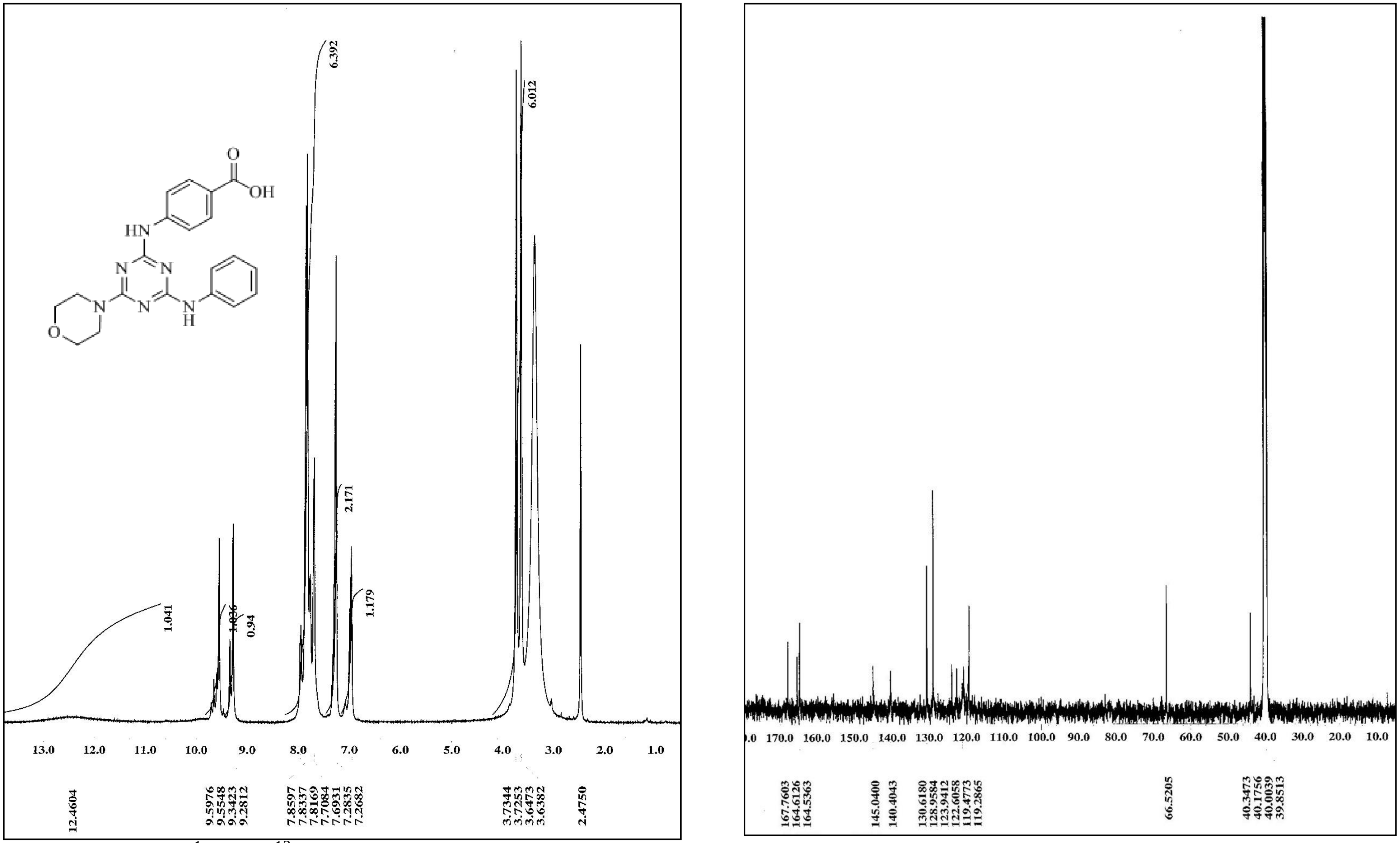

Figure 16: ^{1}H- and ^{13}C-NMR (500 MHz, DMSO-d_6) spectrum of 4-((4-morpholino-6-(phenylamino)-1,3,5-triazin-2-yl)amino)benzoic acid **19.**

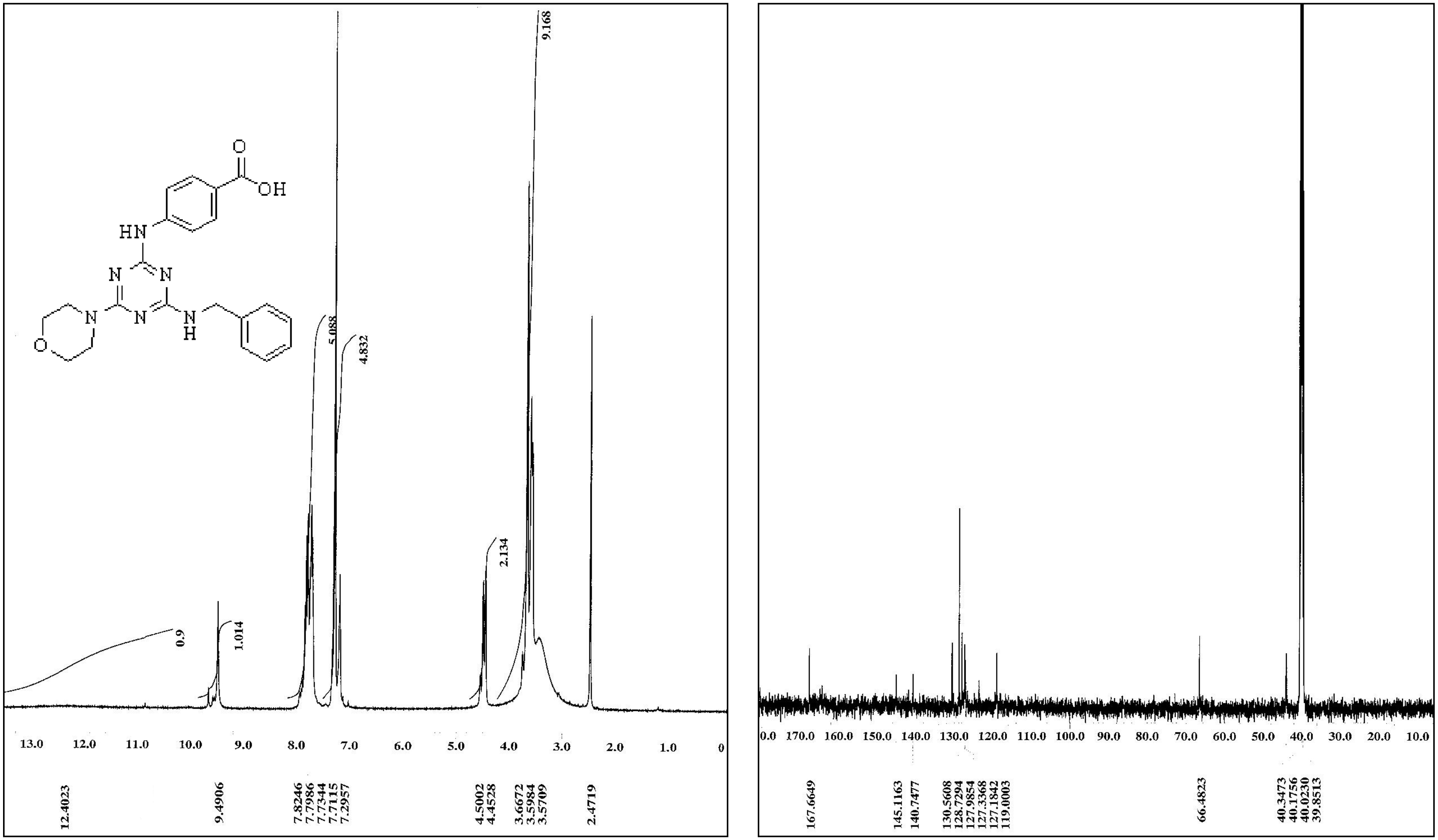

Figure 17: ^{1}H- and ^{13}C-NMR (500 MHz, DMSO-d_6) spectra of 4-((4-(benzylamino)-6-morpholino-1,3,5-triazin-2-yl)amino)benzoic acid **20.**

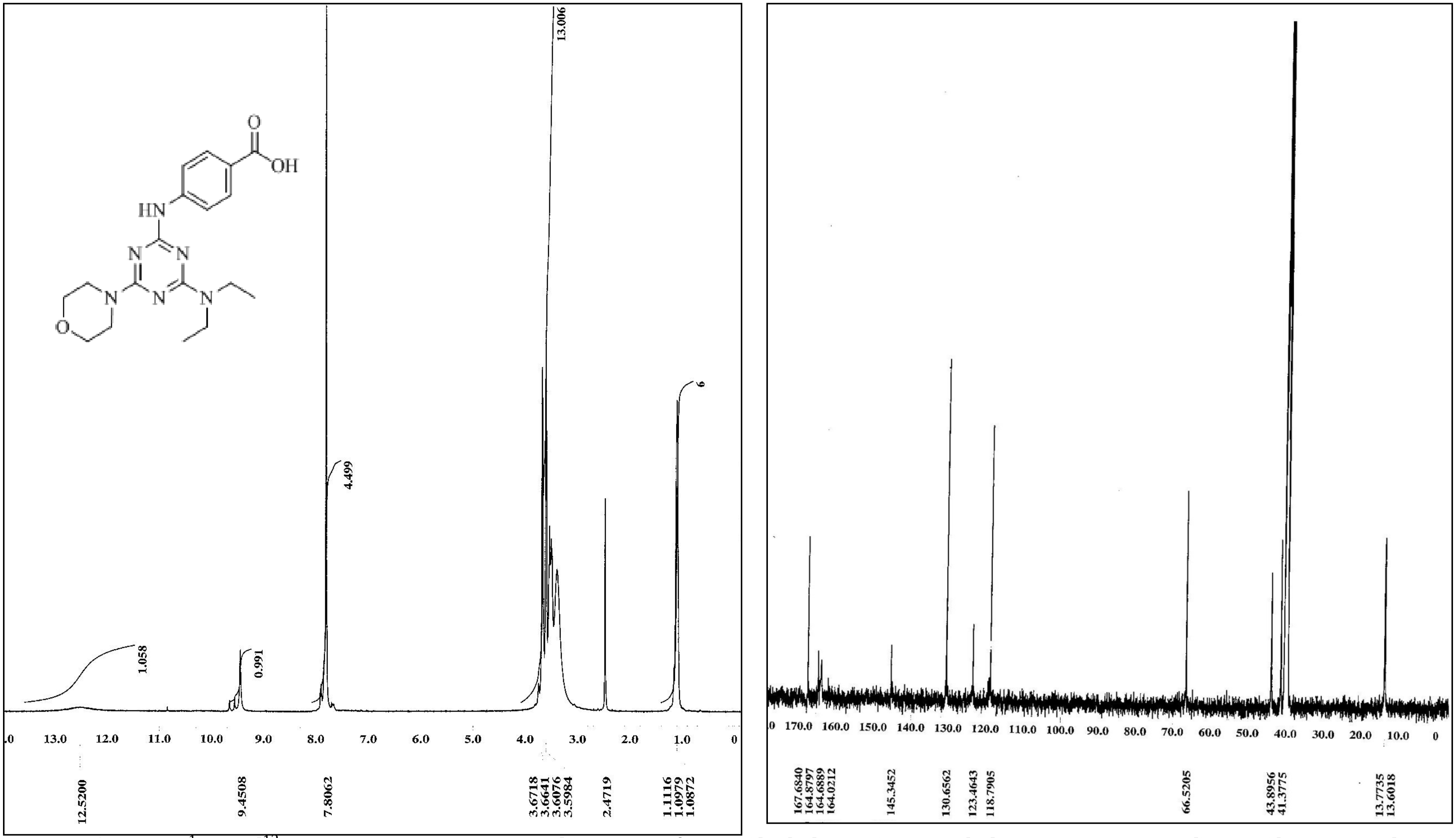

Figure 18: ^{1}H- and ^{13}C-NMR (500 MHz, DMSO-d_6) spectra of 4-((4-(diethylamino)-6-morpholino-1,3,5-triazin-2-yl)amino)benzoic acid **21.**

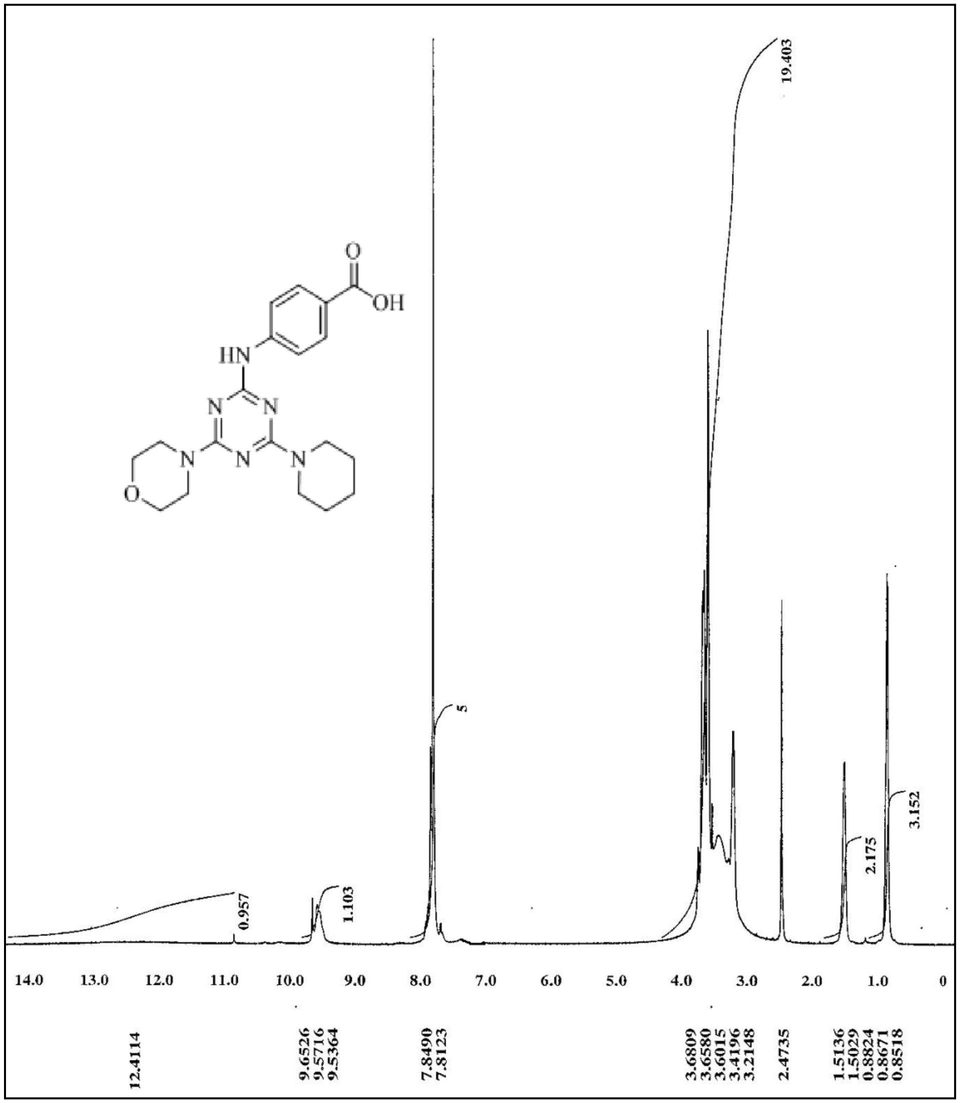

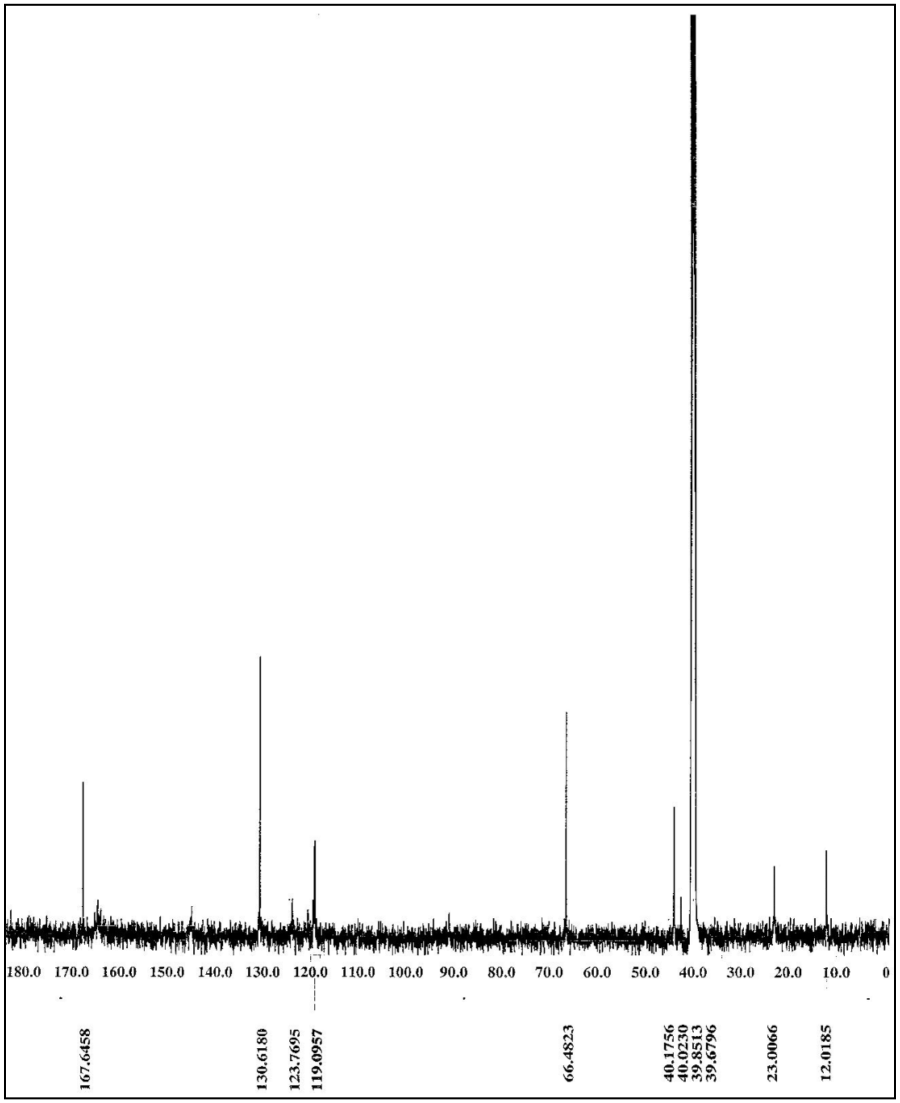

Figure 19: ^{1}H- and ^{13}C-NMR (DMSO-d_6) spectra of 4-((4-morpholino-6-(piperidin-1-yl)-1,3,5-triazin-2-yl)amino)benzoic acid **22.**

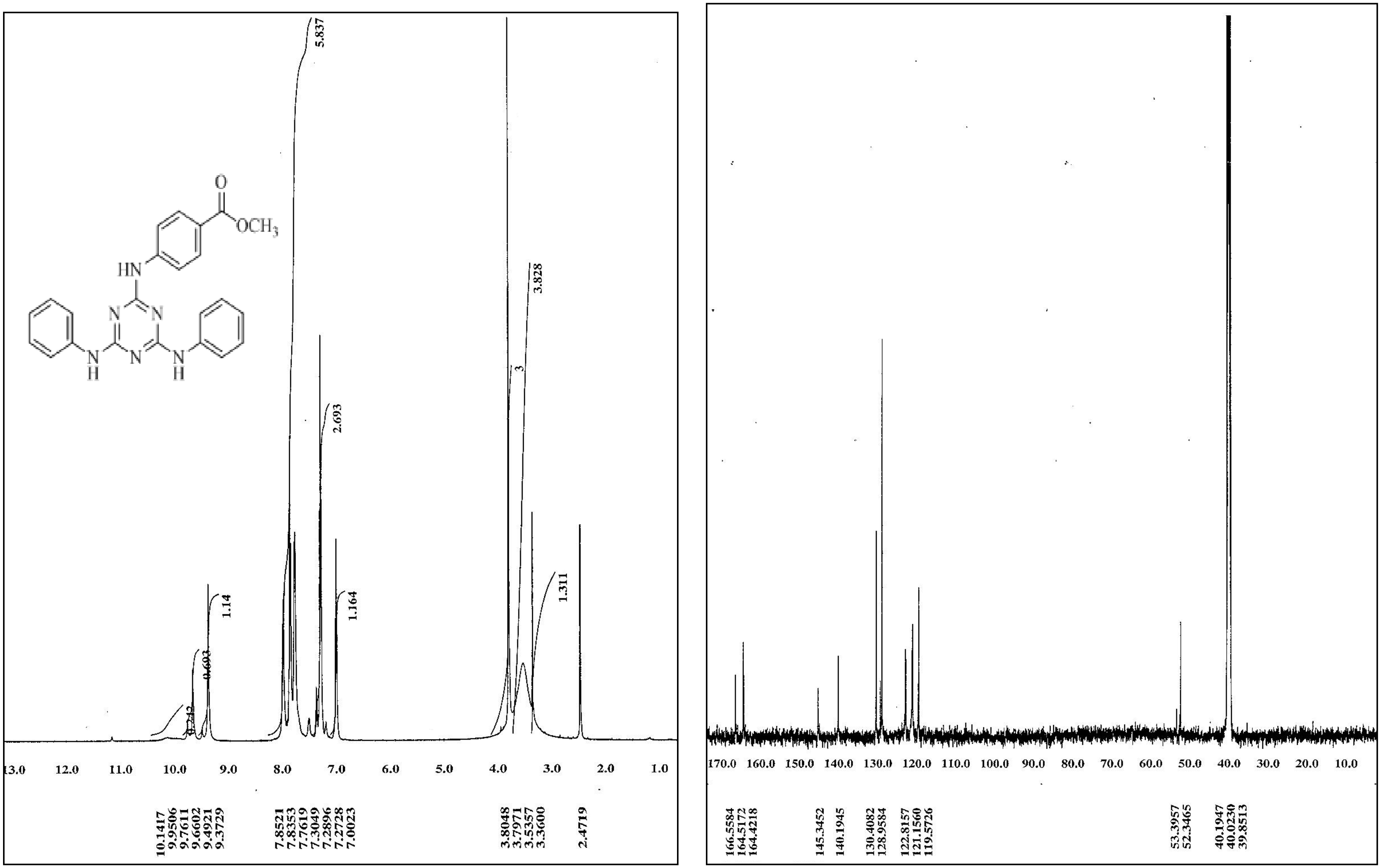

Figure 20:^{1}H-NMR (500 MHz, DMSO-d_6) spectrum of methyl N-(4,6-Dianilino-1,3,5-triazin-2-yl)amino benzoate **23.**

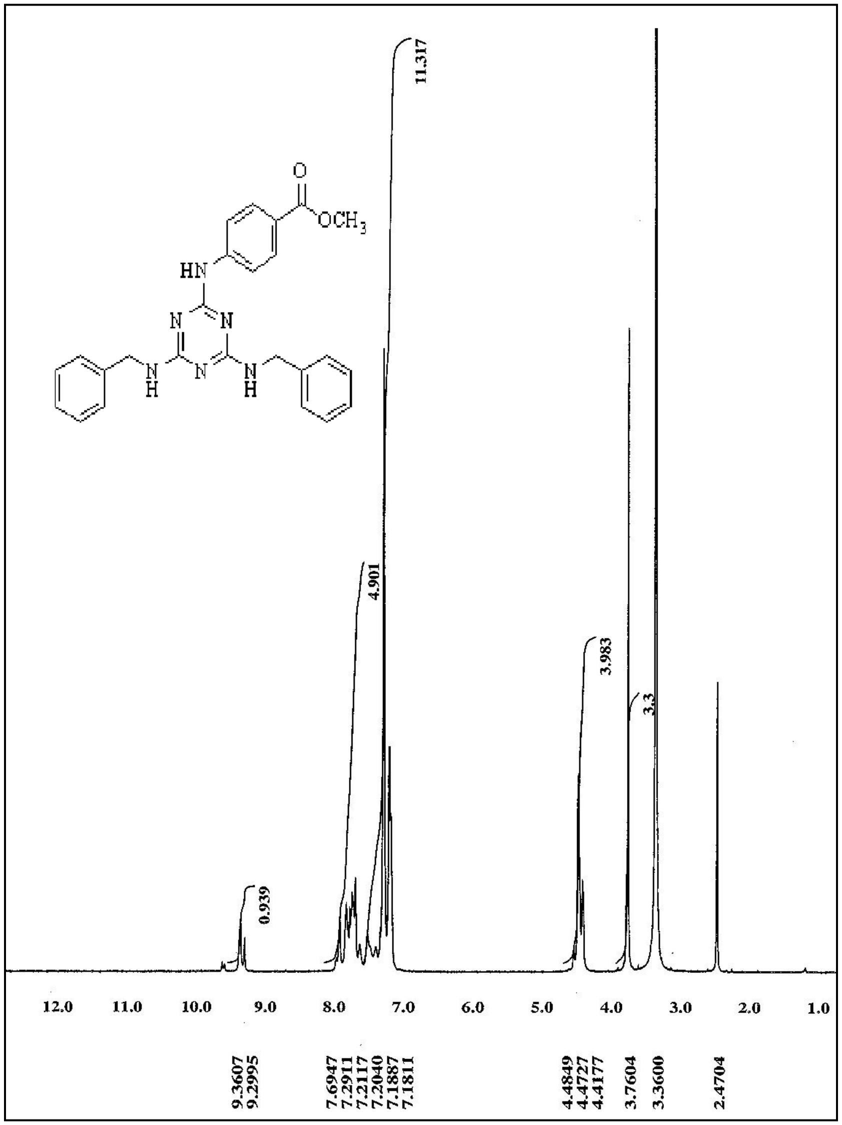

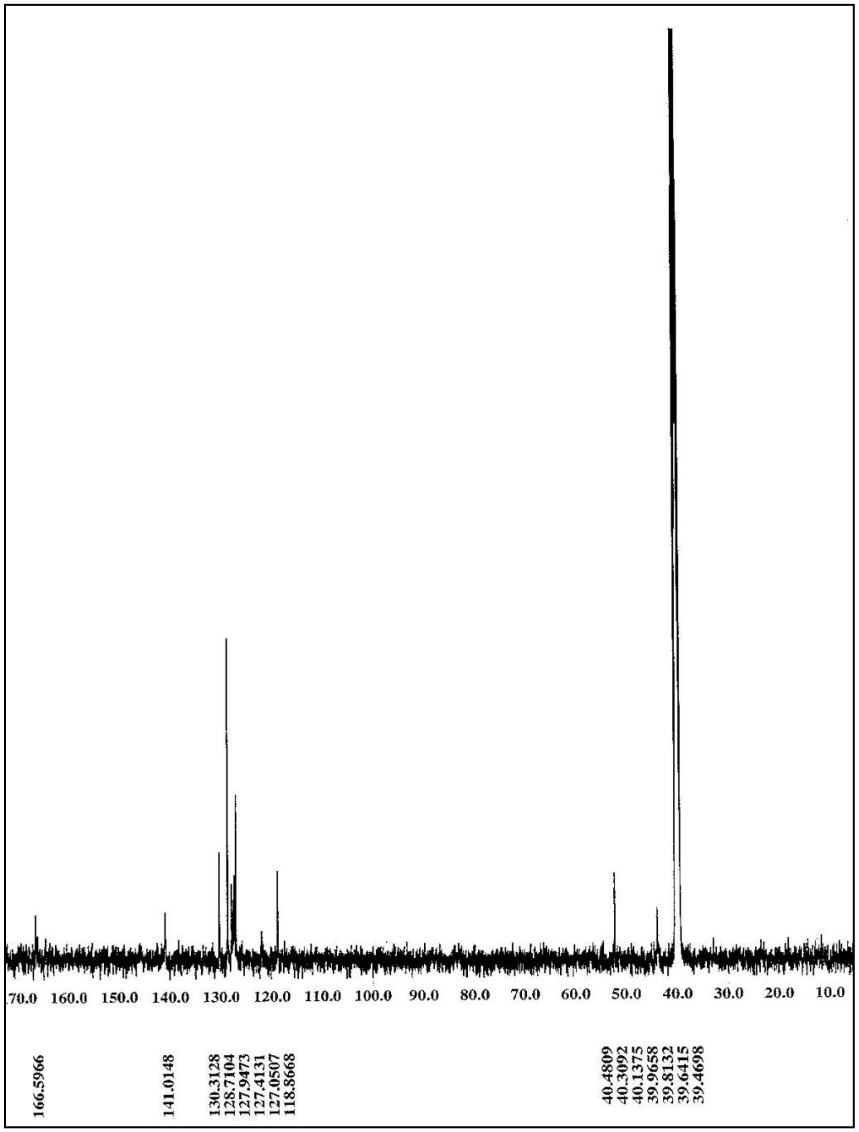

Figure 21:^{1}H- and ^{13}C-NMR (DMSO-d_6) spectra of methyl 4-((4,6-bis(benzylamino)-1,3,5-triazin-2-yl)amino)benzoate **24.**

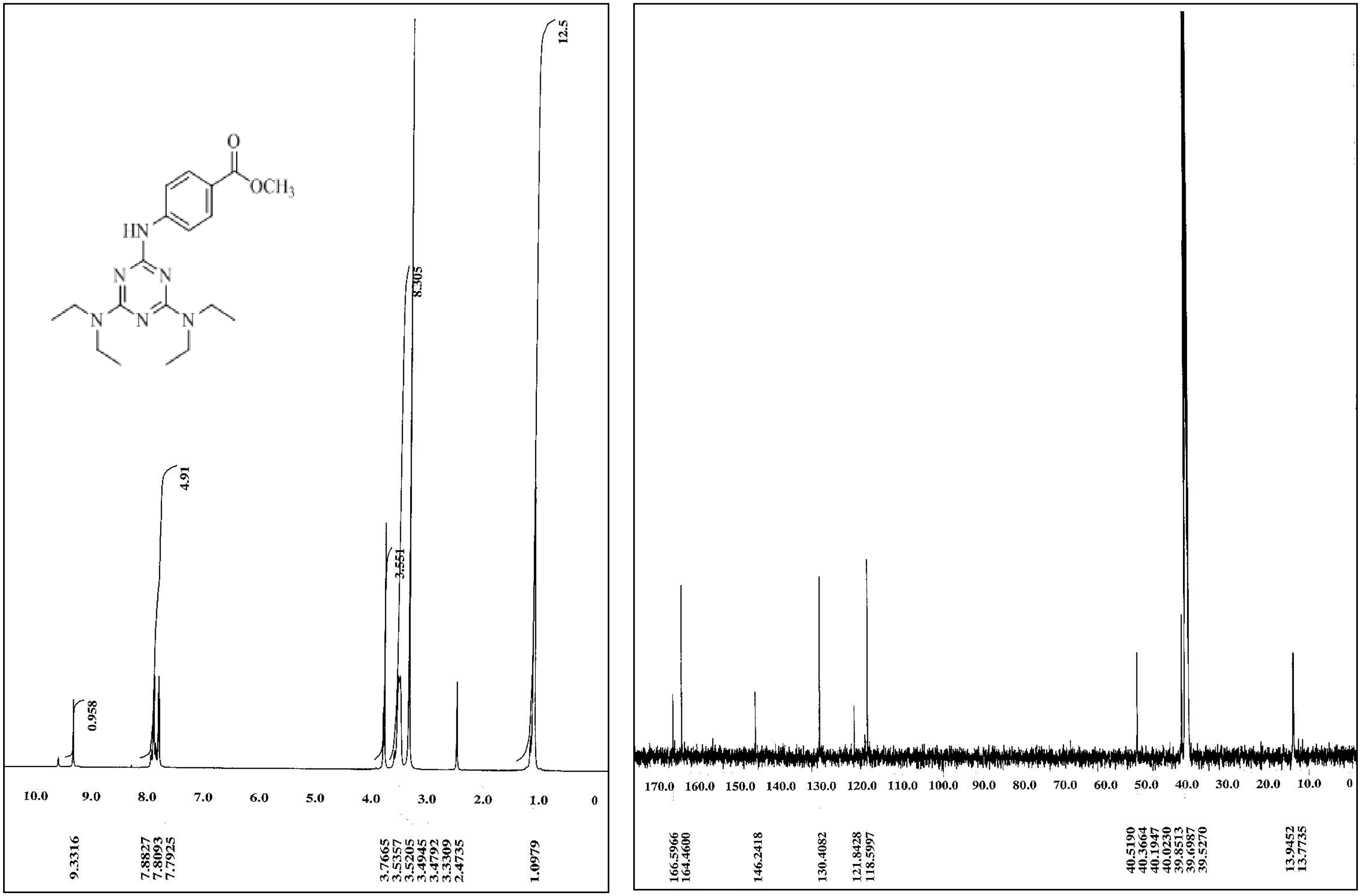

Figure 22: ^{1}H- and ^{13}C-NMR (500 MHz, DMSO-d_6) spectra of methyl 4-((4,6-bis(diethylamino)-1,3,5-triazin-2-yl)amino)benzoate **25.**

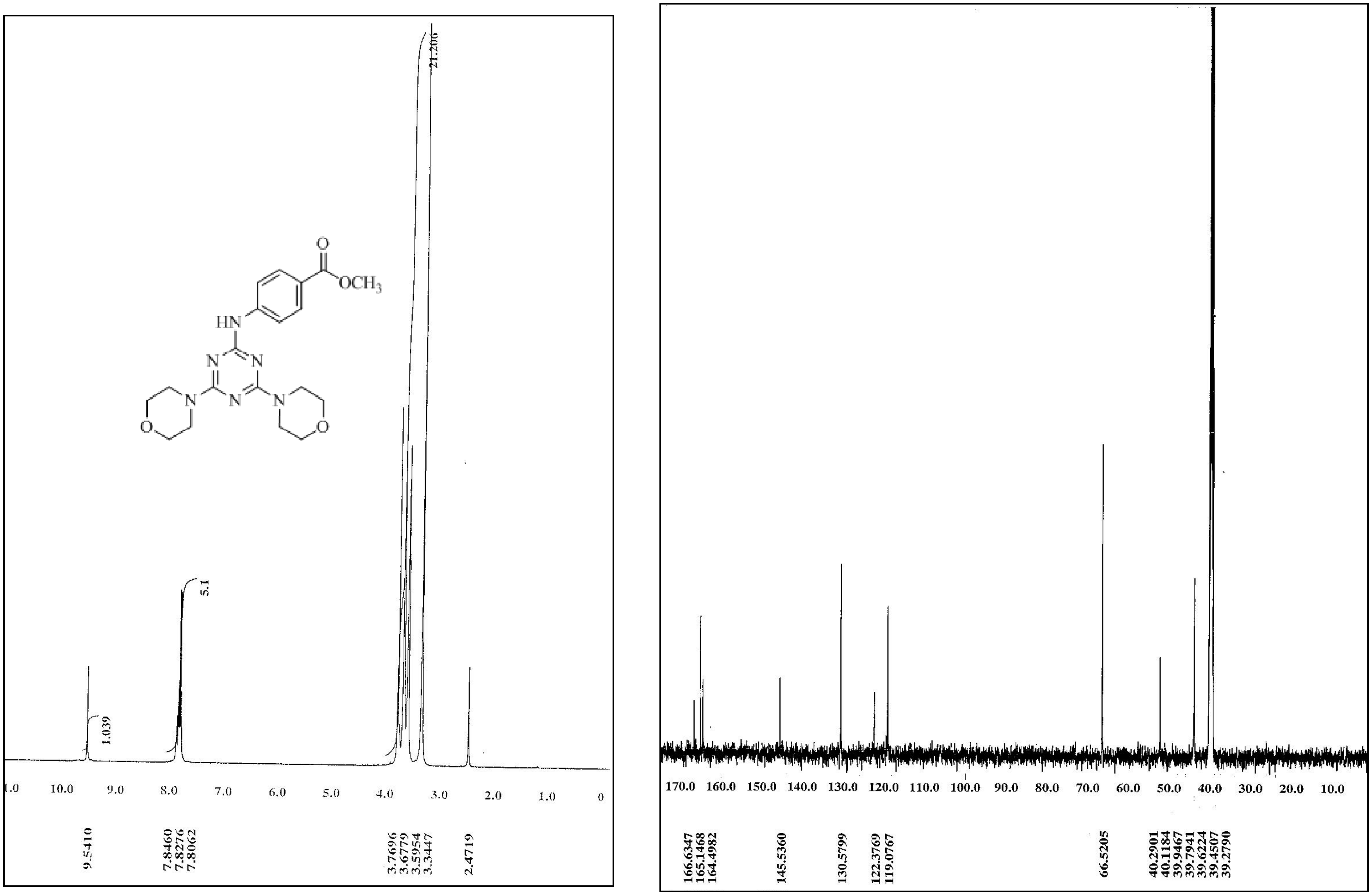

Figure 23: ^{1}H- and ^{13}C-NMR (500 MHz, DMSO-d$_6$) spectra of methyl 4-((4,6-dimorpholino-1,3,5-triazin-2-yl)amino)benzoate **26.**

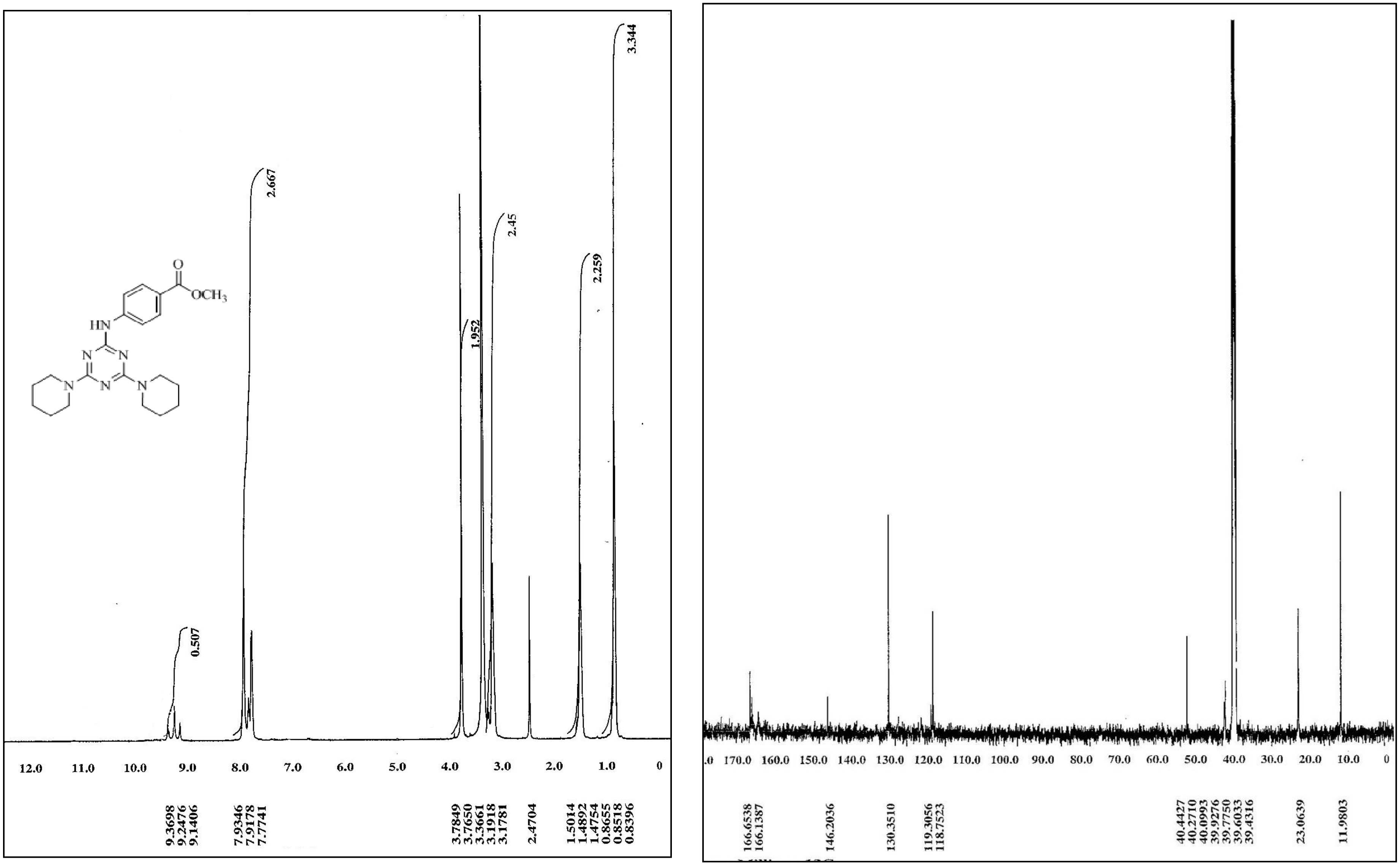

Figure 24: ^{1}H- and ^{13}C-NMR (500 MHz, DMSO-d_6) spectra of methyl 4-((4,6-di(piperidin-1-yl)-1,3,5-triazin-2-yl)amino)benzoate **27.**

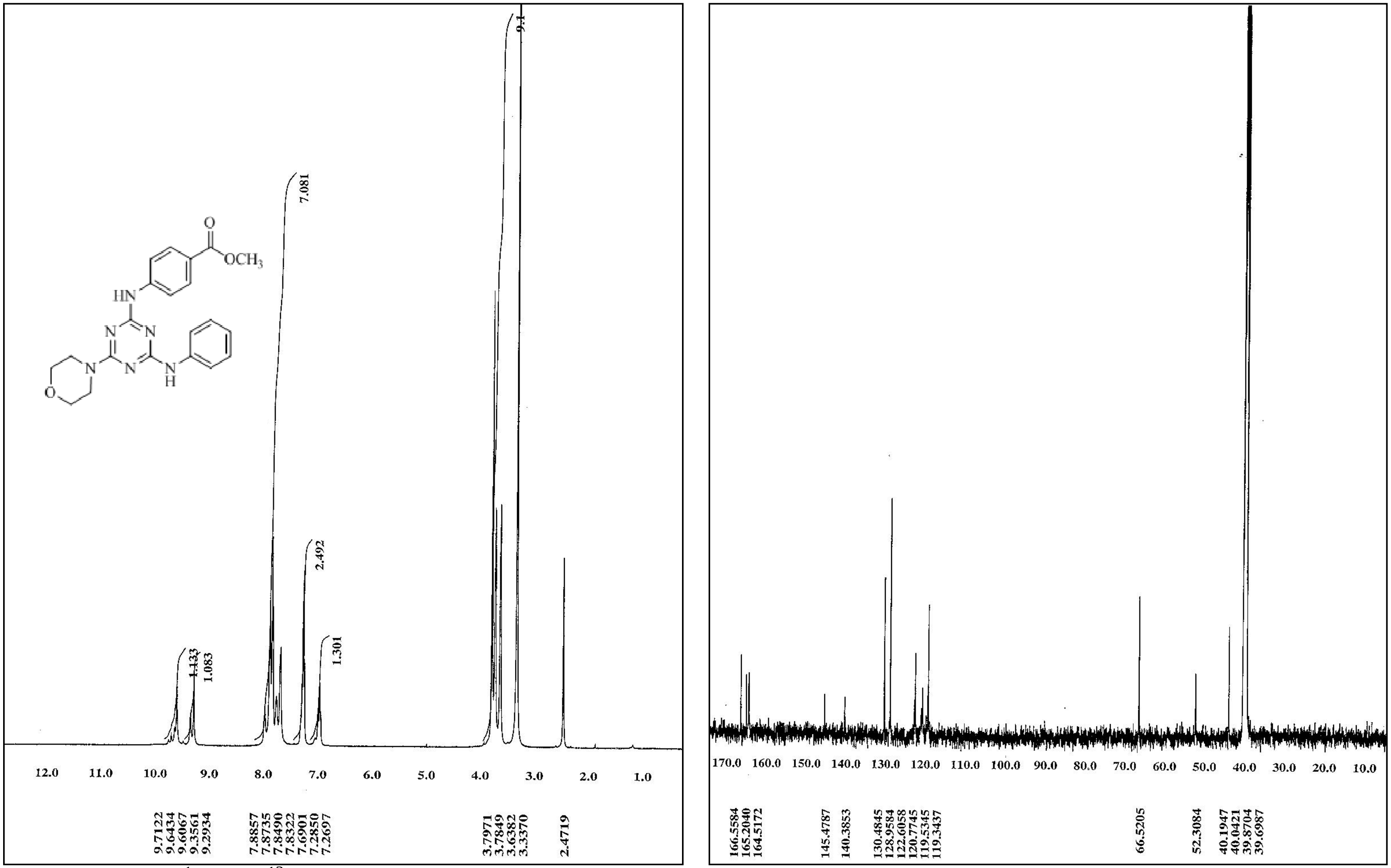

Figure 25: ^{1}H- and ^{13}C-NMR (500 MHz, DMSO-d$_6$) spectra of methyl 4-((4-morpholino-6-(phenylamino)-1,3,5-triazin-2-yl)amino)benzoate **28.**

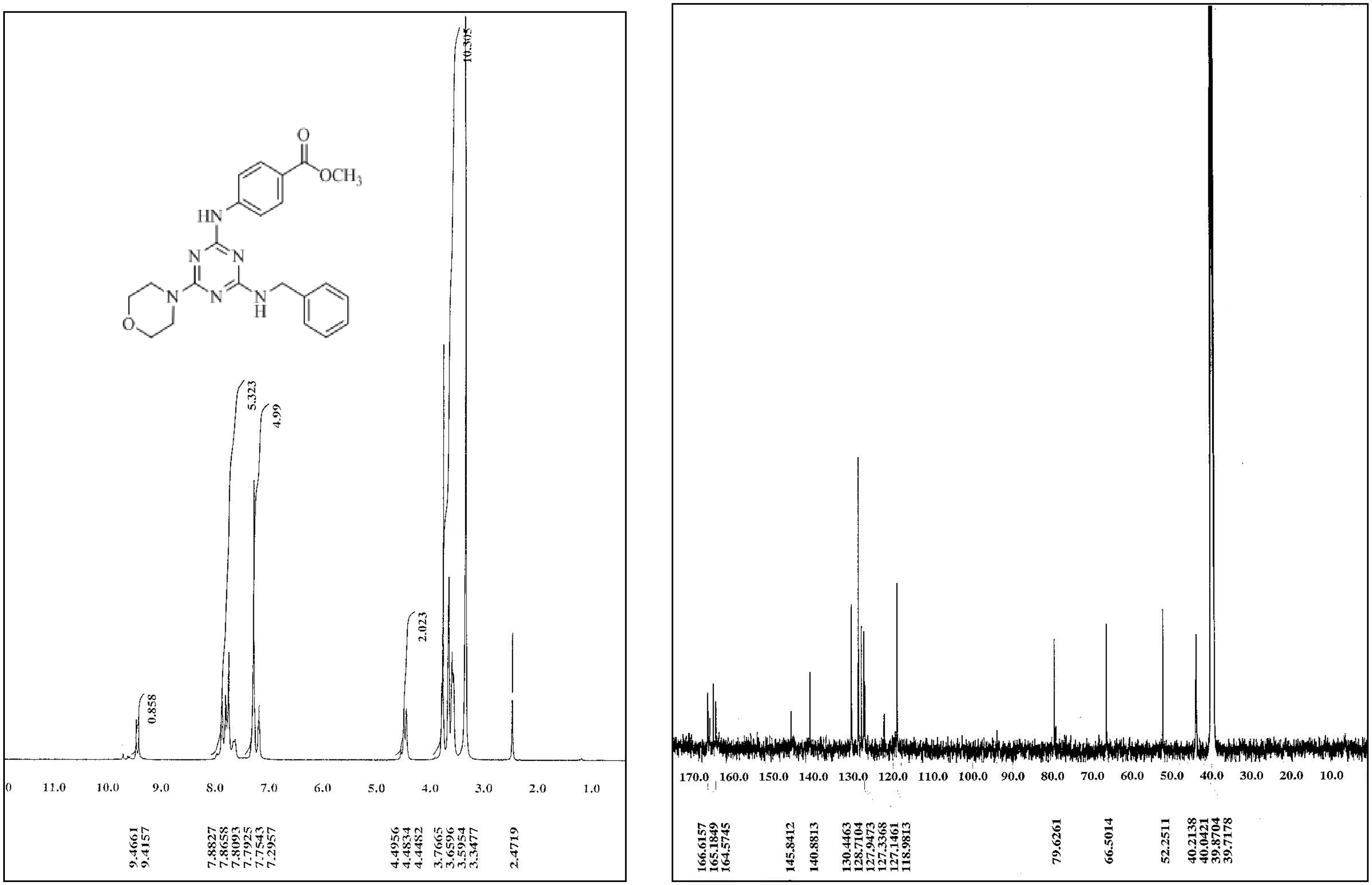

Figure 26: ^{1}H-and^{13}C-NMR (500 MHz, $CDCl_3$) spectra of methyl 4-((4-(benzylamino)-6-morpholino-1,3,5-triazin-2-yl)amino)benzoate **29.**

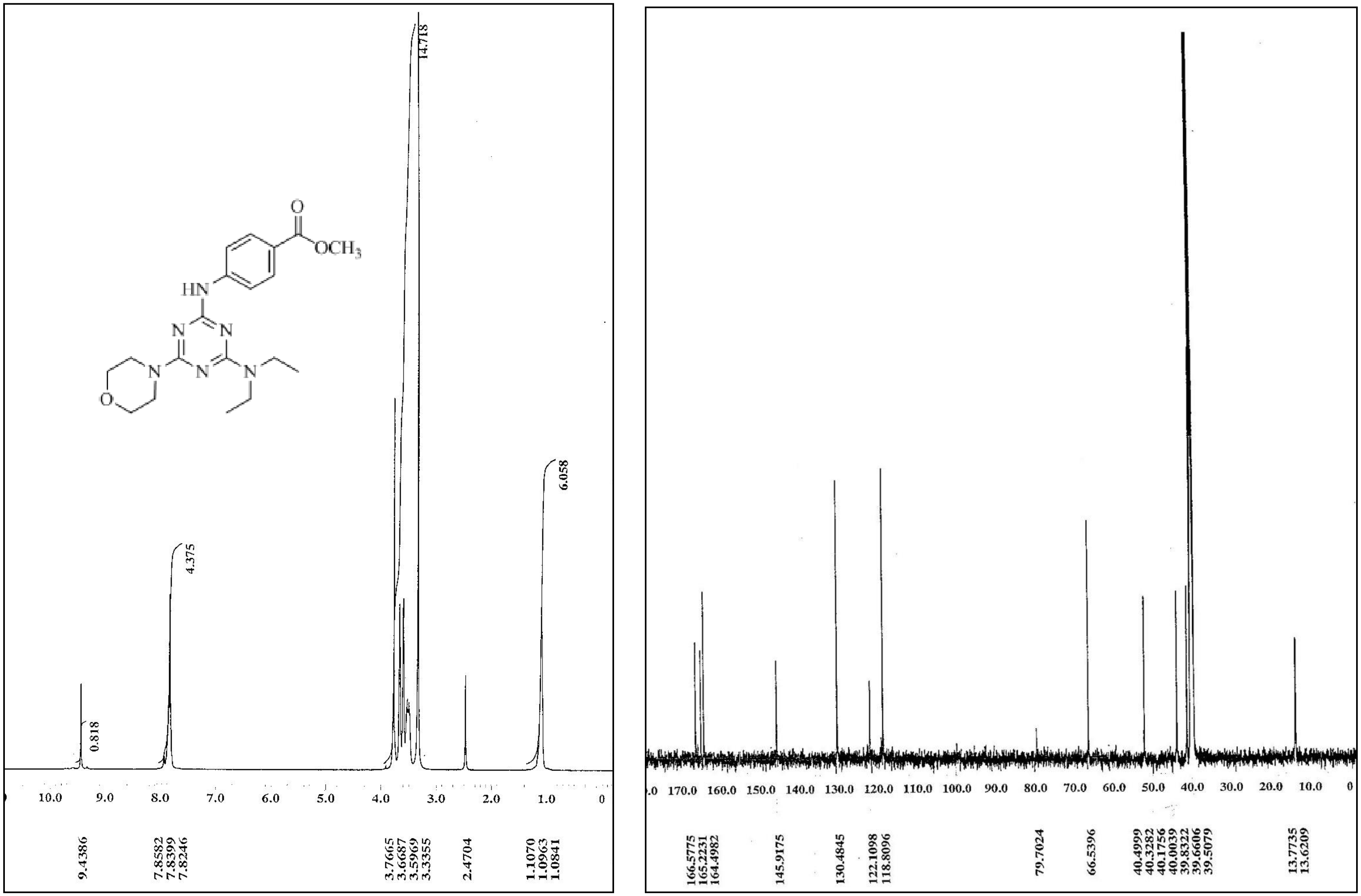

Figure 27: ^{1}H-and^{13}C-NMR (500 MHz, $CDCl_3$) spectra of methyl 4-((4-(diethylamino)-6-morpholino-1,3,5-triazin-2-yl)amino)benzoate **30.**

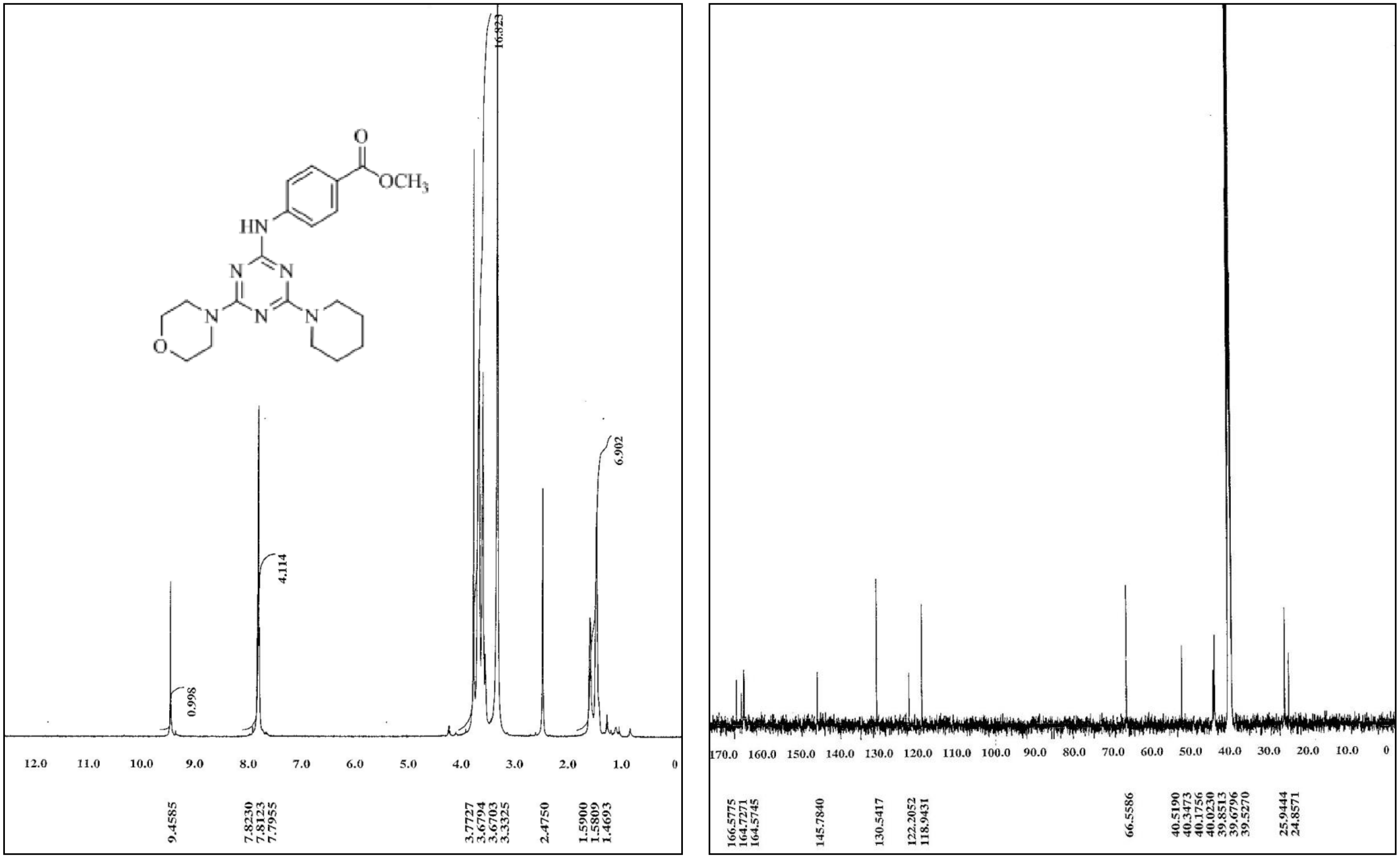

Figure 28: ^{1}H-and^{13}C-NMR (500 MHz, $CDCl_3$) spectra of methyl 4-((4-morpholino-6-(piperidin-1-yl)-1,3,5-triazin-2-yl)amino)benzoate **31.**
